# MyoMiner: explore gene co-expression in normal and pathological muscle

**DOI:** 10.1186/s12920-020-0712-3

**Published:** 2020-05-11

**Authors:** Apostolos Malatras, Ioannis Michalopoulos, Stéphanie Duguez, Gillian Butler-Browne, Simone Spuler, William J. Duddy

**Affiliations:** 1 Sorbonne Université, Inserm, Institut de Myologie, U974, Center for Research in Myology, 47 Boulevard de l’hôpital, 75013 Paris, France; 2 Centre of Systems Biology, Biomedical Research Foundation, Academy of Athens, 4 Soranou Ephessiou St., 11527 Athens, Greece; 3 Northern Ireland Centre for Stratified Medicine, Biomedical Sciences Research Institute, C-TRIC, Altnagelvin Hospital Campus, Glenshane Road, Ulster University, Derry/Londonderry, BT47 6SB UK; 4 Muscle Research Unit, Experimental and Clinical Research Center – a joint cooperation of the Charité Medical Faculty and the Max Delbrück Center for Molecular Medicine, Lindenberger Weg 80, 13125 Berlin, Germany

**Keywords:** Transcriptomics, Correlation, Gene co-expression, Gene co-expression networks, Differential correlation, Functional genomics

## Abstract

**Background:**

High-throughput transcriptomics measures mRNA levels for thousands of genes in a biological sample. Most gene expression studies aim to identify genes that are differentially expressed between different biological conditions, such as between healthy and diseased states. However, these data can also be used to identify genes that are co-expressed within a biological condition. Gene co-expression is used in a guilt-by-association approach to prioritize candidate genes that could be involved in disease, and to gain insights into the functions of genes, protein relations, and signaling pathways. Most existing gene co-expression databases are generic, amalgamating data for a given organism regardless of tissue-type.

**Methods:**

To study muscle-specific gene co-expression in both normal and pathological states, publicly available gene expression data were acquired for 2376 mouse and 2228 human striated muscle samples, and separated into 142 categories based on species (human or mouse), tissue origin, age, gender, anatomic part, and experimental condition. Co-expression values were calculated for each category to create the MyoMiner database.

**Results:**

Within each category, users can select a gene of interest, and the MyoMiner web interface will return all correlated genes. For each co-expressed gene pair, adjusted *p*-value and confidence intervals are provided as measures of expression correlation strength. A standardized expression-level scatterplot is available for every gene pair r-value. MyoMiner has two extra functions: (a) a network interface for creating a 2-shell correlation network, based either on the most highly correlated genes or from a list of genes provided by the user with the option to include linked genes from the database and (b) a comparison tool from which the users can test whether any two correlation coefficients from different conditions are significantly different.

**Conclusions:**

These co-expression analyses will help investigators to delineate the tissue-, cell-, and pathology-specific elements of muscle protein interactions, cell signaling and gene regulation. Changes in co-expression between pathologic and healthy tissue may suggest new disease mechanisms and help define novel therapeutic targets. Thus, MyoMiner is a powerful muscle-specific database for the discovery of genes that are associated with related functions based on their co-expression.

MyoMiner is freely available at https://www.sys-myo.com/myominer

## Background

High-throughput data are crucial for modern biology. cDNA microarrays have provided an efficient way to measure the expression of thousands of genes simultaneously [1, 2], thus helping the study of fundamental biological processes such as gene regulation, signaling pathways and even complex disease traits. The main use of microarrays is differential gene expression analysis where two or more sets of samples are compared (e.g. treated or diseased vs normal) and the up- or down-regulated genes are identified. The accumulation of large amounts of data over the years in public high-throughput data repositories such as ArrayExpress [3] and Gene Expression Omnibus [4], allows us to identify relations between genes through correlation analysis. However, it is difficult for experimental researchers to combine and extract the information they seek if they have limited bioinformatics expertise.

Measures of gene co-expression obtained by the analysis of data stored in high-throughput repositories such as ArrayExpress [3] and Gene Expression Omnibus [4] are now widely used to study gene function, protein relations and biological networks such as signaling pathways [5, 6], and several tools and resources exist that facilitate the exploration and analysis of gene co-expression across tissues, common technological platforms, or conditions [7–9]. Furthermore, pathology-specific gene co-expression can be used as a biomarker discovery tool [10] or for patient prognosis [11, 12].

An important purpose of gene co-expression analysis is in discovering the mechanistic links between genes. Gene co-expression is a form of functional association, alongside other types of functional associations such as protein-protein interactions determined by immunoprecipitation experiments, or protein cellular co-localization as determined by immunostaining. Since functional association data can be regarded as a graph structure, gene co-expression can be used for network biology or network medicine types of analysis [13, 14]. As such, the study of gene co-expression can be used to understand better the mechanisms of molecular interaction within a cell. This may be the whole cell, in which case it can be called interactomics, or it can be focused on a specific gene, function, or pathway. Gene co-expression analysis therefore can enhance the study of changes to molecular interaction networks, and can be applied both at the whole cell level and for specific cellular functions, and to compare between different pathologies and conditions. Recent work suggests that direct causal relationships between genes may be inferable from gene co-expression [15]. The purpose of gene co-expression analysis is distinct from that of differential expression analysis, the purpose of which is to identify differences in individual gene transcript levels between conditions. Differential expression analysis may be combined with functional enrichment testing to detect changes across gene sets (e.g. representing functions, pathways, and cellular components), but it cannot tell us about mechanistic relationships between genes within a given gene set.

Several organism-specific co-expression databases already exist such as the Arabidopsis Co-expression Tool (ACT) [16, 17] and ATTED-II [18] for *Arabidopsis thaliana*, and CoXPRESdb [19], STARNET [20], Genevestigator [21] and Human Gene Correlation Analysis (HGCA) [22] for mammals. They collect gene expression data and a Pearson correlation coefficient [23] is calculated for each pair of genes or probes, which can be used as a measure of expression correlation and for network construction from the highly-correlated genes. However, these databases are not tissue- or cell-specific, because their expression matrices are derived from a mix of tissue types and in some cases from mixed conditions (e.g. treated and untreated cells). Since gene expression differs between types of tissues and cells [24], it is expected that gene co-expression will also vary. Experimentalists seeking to identify correlation patterns for a chosen gene of interest, usually focus on a specific tissue or cell model and thus the relevance of co-expression values is greatly enhanced by the specificity of the data used [25]. ImmuCo [26] and Immuno-Navigator [27] gene co-expression databases are among the first to address immune cell-specific correlation, and the latter also corrects the expression matrices for batch effects. Many conditions, such as reagents, equipment, software and personnel could vary during the course of an experiment and may introduce batch effects, which is a common and strong source of variation on high-throughput data [28, 29]. Batch effects are unrelated to biological or scientific variables, are not corrected by normalization [29] and must be removed before any further analysis. By combining studies, one extra layer of batch effects is introduced: experiments from different laboratories [30]. If left uncorrected, this technical variation will introduce error into the results of correlation analysis. Another issue is that current co-expression databases include gene co-expression from healthy samples only or from a mix of healthy and diseased conditions. Studying the changes in co-expression between healthy and pathological states could lead to biomarker discovery and to improved understanding of disease mechanisms [31].

Here, we introduce MyoMiner (https://www.sys-myo.com/myominer), the first striated muscle cell- and tissue-specific database that provides co-expression analyses in both normal and pathological tissues, addressing both issues of overall correlation and batch effects. MyoMiner includes 2376 mouse and 2228 human microarray samples separated in 142 human, mouse and cell categories based on age, sex, anatomic part and condition. We built a simple and easy-to-use web interface to search for transcriptional correlation of any expressed gene pair in muscle cells/tissues and the various pathological conditions. Users can select a category and a gene of interest, and MyoMiner will return all the expressed correlated genes for that category. Correlation strength is measured by the provided FDR adjusted *p*-value (q-value) and confidence intervals are given for each correlation.

## Construction and content

### Microarray data collection

To collect muscle-specific microarray data and discard low quality samples, we followed a pipeline similar to that used for our Muscle Gene Sets resource [32], as described below. Even though ArrayExpress partially mirrors Gene Expression Omnibus, we searched both repositories for striated muscle (skeletal and cardiac), cells and cell line experiments. In this initial screening, we found that the most popular platforms used for muscle-related experiments were Affymetrix Human Genome U133 Plus 2.0 GeneChip (GEO platform GPL570 or ArrayExpress ID A-AFFY-44) for human and Affymetrix Mouse Genome 430 2.0 GeneChip (GEO platform GPL1261 or ArrayExpress ID A-AFFY-45) for murine samples. Since correlation analysis requires homogenous data, we limited our more refined subsequent searches to these two platforms, which represent about half of all muscle data on both repositories.

We searched ArrayExpress using the following strings: *(muscle(s) OR myoblast(s) OR myotube(s) OR myofiber(s) OR cardiomyocyte(s) OR myocyte(s) OR heart(s) OR HSMM) AND A-AFFY-44* for human samples, and *(muscle(s) OR myoblast(s) OR myotube(s) OR myofiber(s) OR cardiomyocyte(s) OR myocyte(s) OR heart(s) OR C2C12 OR HL1 OR G8 OR SOL8) AND A-AFFY-45* for murine samples. GEO and ArrayExpress assign a different ID to each alternative platform. An alternative platform is the same microarray chip as the original, but the data are pre-processed with a different probe-to-gene mapping file called Chip Description File (CDF). It is quite popular for researchers to use a different CDF than the original for better probe-to-probeset and probeset-to-gene targeting accuracy (see “Probes to gene mapping” section). GEO provides a list of the alternative platforms into the original platform information sheet, but many were missing. An additional way to identify the alternative platforms is to search on ArrayExpress (which is manually curated) for alternative IDs. In the array browser of ArrayExpress (https://www.ebi.ac.uk/arrayexpress/arrays/browse.html), we searched for U133 Plus 2.0, MG 430 2.0 and retrieved all of the alternative GEO platforms and IDs to A-AFFY-44 [GEO: GPL570] for human and to A-AFFY-45 [GEO: GPL1261] for mouse (Additional file 1: Table S1).

Next, we developed a script to parse automatically their MIAME [33] metadata and confirm them manually, selecting only those pertinent to muscle research. We excluded all series that did not include the raw CEL files (Affymetrix fluorescence light intensity files) because we pre-processed them using a robust data analysis pipeline, described in detail below, so as to homogenize the data as much as possible.

Particular microarray samples may have been used for several experiments, or analyzed with different normalization algorithms, or even grouped with other samples in larger meta-analyses, the results of which have been re-submitted to the repositories. The reused microarrays get a different ID (GSM number in GEO) and it is crucial to identify and remove them from co-expression analysis, as duplicates will erroneously increase correlation scores and introduce biases. Using the conversion tool (apt-cel-convert.exe) of Affymetrix Power Tools [34], we transformed the binary CEL files (version 4) to ASCII text format (version 3) in order to parse them. Their light intensity values were concatenated into a string and used as input to three hash algorithms: MD5 [35], SHA-1 [36] and CRC32 [37]. The combined hash acts as a unique key for each sample and the duplicate arrays were then easily identified and removed.

### Quality control of Affymetrix microarrays

The quality control pipeline was identical to that used previously for our Muscle Gene Sets resource [32]. Arrays that had extreme values or were above our set thresholds on the combined quality controls, were excluded from any further analysis. In total, we removed 160 human and 122 mouse samples (Additional file 1: Table S2, S3). We identified the poor quality arrays based primarily on the output of percent present, RLE and NUSE, as they are known to perform well [38], and secondarily on GAPDH and β-actin ratios.

### Data normalization

Pre-processing algorithms, usually termed normalization algorithms, are three-step processes: background correction, normalization and probe summarization. An additional optional step is log_2_ transformation. The arrays that passed quality controls were pre-processed with the Single Channel Array Normalization (SCAN) algorithm [39] with default parameters except for the CDFs, which were downloaded from BrainArray Ensembl ENSG version 20.0.0 [40]. SCAN normalizes each array independently from its series, corrects GC bias and reduces probe and array variation from each individual sample while increasing signal-to-noise ratio. Single array normalization is preferred when combining microarray samples from different series or laboratories, because other pre-processing algorithms such as RMA [41] or GC-RMA [42] use information across samples for both normalization and summarization steps, and can thus introduce correlation artifacts [43, 44].

### Probes-to-genes mapping

At the time of chip design, Affymetrix selection of probes relied on early genome and transcriptome annotation which is significantly different from our current knowledge. The genes on the microarray chips are usually represented by multiple probesets and, conversely, in many cases, a single probeset could target multiple genes or even no gene. Multiple probesets targeting the same gene could exhibit wildly different expression levels making downstream analysis challenging. This limitation had been observed [40], and BrainArray portal was created to reorganize probesets with up-to-date genomic, cDNA and single nucleotide polymorphism (SNP) information in order to create a more accurate and precise CDF. This approach has become very popular amongst researchers [45]. BrainArray CDFs are annually updated and many microarray algorithms and tools now use them by default. The SCAN normalization algorithm has in-built parameters to download and use BrainArray CDFs. For MyoMiner we used Ensembl genome [46] (ENSG) version 20.0.0. We set the SCAN CDF specified parameter *probeSummaryPackage* to *InstallBrainArrayPackage(“human_sample_name.CEL”, “20.0.0”, “hs”, “ensg”)* and *InstallBrainArrayPackage(“mouse_sample_name.CEL”, “20.0.0”, “mm”, “ensg”)* for human and mouse organisms respectively.

### Filtering and mapping of expressed genes to gene symbols

In order to distinguish between expressed and unexpressed genes (such as genes with expression levels close to or lower than the background noise), we used the Universal exPression Code (UPC) algorithm [47] separately for each category. We did that because different tissues, cells or pathological conditions have distinct genetic profiles. UPC is a 2-step algorithm that corrects for background noise using linear statistical models and estimates the percentage of gene expression by calculating the active and inactive gene population. An assumption is made that genes with identical molecular characteristics should share the same background expression levels. To identify expressed genes for each category, we calculated UPC’s percentage expression 3rd quartile for each gene and categorized it as being expressed if its value was higher than 50%.

To map Ensembl gene IDs to HGNC gene symbols [48], Entrez IDs [49] and Uniprot accession numbers [50], we used Ensembl BioMart [51]. We extracted the required information from GRCh38.p5 assembly for human and GRCm38.p4 assembly for mouse.

### Gender prediction

On approximately half of the MIAME metadata entries for both organisms, the gender information was missing [52]. To predict the missing gender entries we used hgfocus.db [53] and mouse4302.db [54] from Bioconductor to map genes to chromosomes and then we calculated the median expression of Y chromosome genes. Males should have higher expression values than females, which was visible on the Y chromosome gene expression histogram with two clearly separated gender related peaks.

### Combining datasets

To define categories of similar samples based on organism, gender, age, anatomic part and condition criteria, we extracted all the available MIAME metadata for each organism (Additional file 2: Table S7 and Additional file 3: Table S8). Then, we filtered all possible metadata combinations into categories that had at least *n* = 12 samples. With this approach we created a large number of categories while maintaining a high level of power. For each category, we created a single expression matrix that includes all the samples from that category. Further analyses such as batch correction and gene co-expression were based on each category’s expression matrix.

### Batch effects evaluation

For batch effect reduction we used the ComBat algorithm [55] from the “SVA” Bioconductor package [56]. ComBat is a robust empirical Bayes method that adjusts for known batch covariates. By default, we considered each data series (i.e. study) to be a different batch for every category (gender, age, etc). However, it is also known that processing date/time can be a strong batch surrogate [29]. From the text converted CEL files we retrieved the scan dates and also used these as batch surrogates for each series, assuming that microarray experiments performed on the same day belonged to the same experimental batch, thus subdividing the aforementioned default series batches to date and series batches. Using principal component analysis (PCA) 3D plots, by the “rgl” R package [57], for each category, we identified if the samples correlate with batch surrogates and proceeded with batch correction if necessary (Table S4). We did not use the category differences as input for the ComBat algorithm (*modcombat = model.matrix(~ 1,numbatches)*), because (a) all samples were from the same category and (b) samples that are assigned to a batch are usually unevenly distributed which can induce incorrect differences [58]. In some cases, when a batch was represented by a single sample, after assessing the PCA 3D plot we assigned the sample to the closest batch cluster if possible, otherwise we used the *mean.only = TRUE* parameter in ComBat that corrects only the mean of the batch effect not adjusting for scale. There were no significant changes (t-test < 0.05, multiple testing controlled with FDR) in gene expression of any gene in any category before and after applying batch correction.

### Gene expression correlation

Spearman’s rank correlation [59] is a non-parametric rank statistic that measures the strength of a monotonic, linear or non-linear, relationship between two sets of data. Monotonic is a function that increases when its independent variable increases, having a positive correlation. If the independent variable decreases while the function increases, the correlation will be negative. Spearman’s correlation is simply the application of Pearson’s correlation [60] on rank converted data. A faster method to calculate Spearman’s *ρ* is to rank the values of *x*_*i*_ and *y*_*i*_, and calculate their difference *d*_*i*_. The rank correlation can then be computed as follows:

1 $$ \rho =1-\frac{6{\sum}_{i=1}^n{d}_i^2}{n\left({n}^2-1\right)} $$

where *n* is the number of samples and *d*_*i*_ *= rank(x*_*i*_*) - rank(y*_*i*_*)*. Spearman’s correlation range values between − 1 and + 1, where − 1 describes a perfect monotonically negative correlation and + 1 a perfect monotonically positive correlation. If the data are monotonically independent, Spearman’s *ρ* is equal to 0. However, this does not necessarily mean that the data are independent in other ways.

Since Spearman’s correlation can be asymptotically approximated by a *t*-distribution with *n-2* degrees of freedom under the null hypothesis of no correlation, we used Student’s *t*-test to examine whether a correlation was significantly different from the null hypothesis:

2 $$ t=\rho \frac{\sqrt{n-2}}{\sqrt{1-{\rho}^2}} $$

To adjust for multiple testing we used the Benjamini – Hochberg (BH) method [61] to control the false discovery rate (FDR). Spearman correlation *ρ-* and adjusted *p*-values were computed with the “psych” R package [62].

Because the correlation coefficient is not distributed normally and its variance is dependent on both sample size and the correlation coefficient from the entire population, we cannot compute confidence intervals directly for the *ρ*-values [63]. First we have to convert *ρ-*values into additive quantities with *ρ* to *Z* Fisher transformation [64] which is the inverse hyperbolic tangent function (arctanh) (Additional file 1: Table S6, Eq. S1, S2).

Second, we compute the confidence intervals at 95% confidence level *Z*_*table*_ = 1.96 (Additional file 1: Table S6, Eq. S3). The final step is to convert *Z* scores back to *ρ*-values using the hyperbolic tangent function (tanh) (Additional file 1: Table S6, Eq. S4).

Thus, in any sample correlation coefficient *ρ*, there is a 95% probability that the true population correlation coefficient value will be in the range of *CI*_*lower*_ and *CI*_*upper*_.

### Differential co-expression

For comparing whether any two correlation coefficients *ρ*_*1*_ and *ρ*_*2*_, for different categories (various samples and sample sizes *n*_*1*_ and *n*_*2*_), are significantly different, we make the null hypothesis (*H*_*0*_) that the correlation coefficients are not statistically different. Then we transform the *ρ*-values to *Z* scores (Additional file 1: Table S6, Eq. S1), calculate the difference between them and calculate an absolute *Z* score by dividing the difference with the pooled standard error:

3 $$ {Z}_c=\left|\frac{Z_1-{Z}_2}{SE_{zp}}\right|,\mathrm{where}\kern0.5em {SE}_{zp}=\sqrt{\frac{1}{n_1-3}+\frac{1}{n_2-3}} $$

If *Z*_*c*_ *< Z*_*table*_ where *Z*_*table*_ *= 1.96* (at 95% confidence level) or more commonly, if the *p*-value which is the probability *P (Z*_*c*_ *< 1.96) > 0.05*, we cannot reject *H*_*0*_. The difference between *ρ*_*1*_ and *ρ*_*2*_ is not significant at 95% confidence level.

### Data extraction for validation

To validate the findings of MyoMiner we compared it to the existing databases MEM, SEEK and the GTEx RNA-Seq collection. Since the databases include generic co-expression data and not specific categories, we limited their studies to muscle relevant as follows: for MEM we selected the GPL570 (U133 Plus 2.0) platform, the Pearson distance method, betaMEM as the ranking method and the dataset filter Stdev = 0 with “*Skeletal Muscle*” as the text field search. For SEEK we used the refined search option to Muscle (Non-cancer) datasets and the Pearson distance method. For GTEx v8 we downloaded the annotation data and extracted the gene TPMs for muscle relevant samples using the options SMTSD =” *Muscle – Skeletal*” and SMAFRZE =” *RNASEQ*”. We then calculated Spearman *ρ* the same way as for MyoMiner.

### Database construction and website implementation

**Fig. 1:**
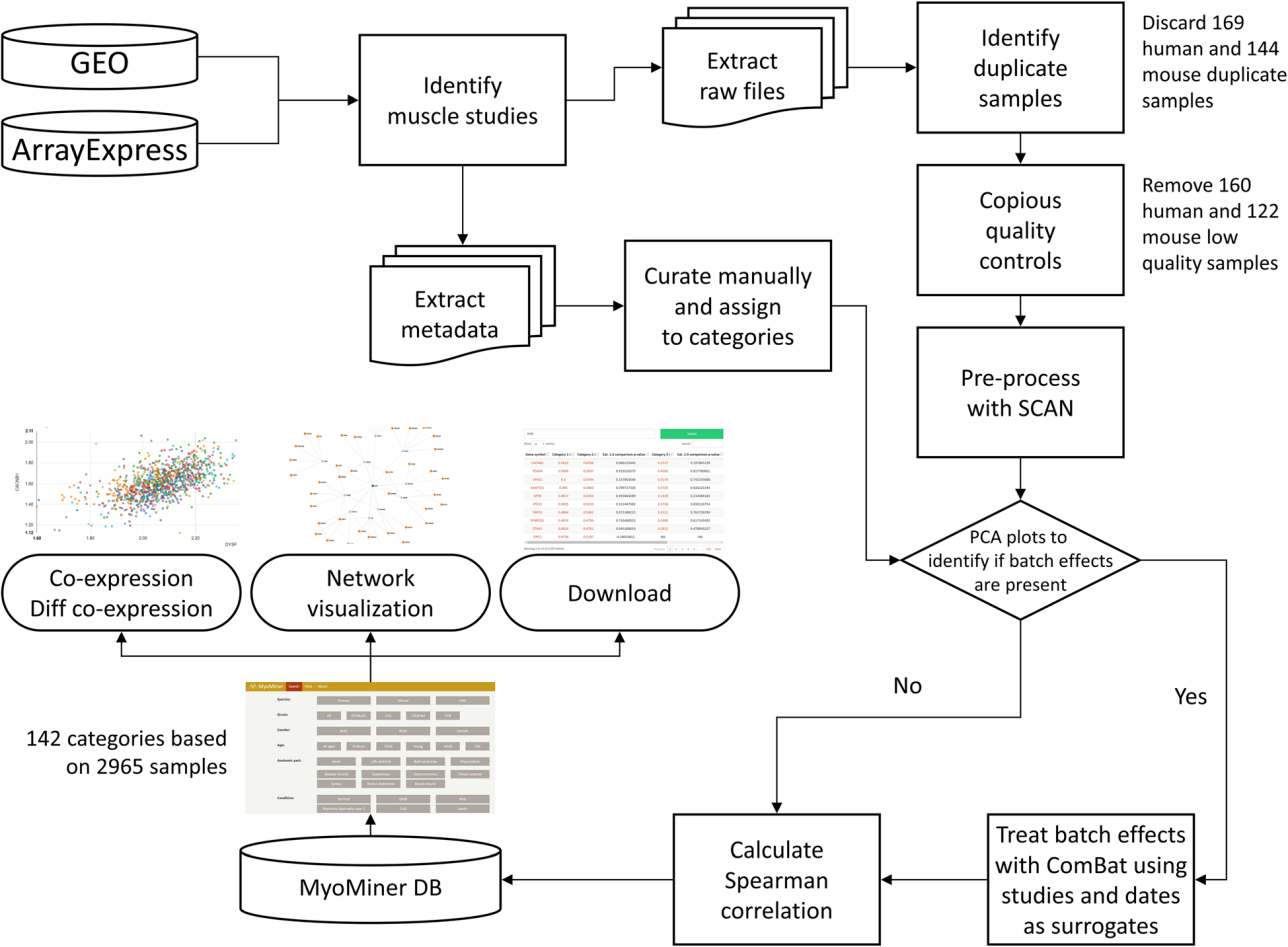
Workflow of data pre-processing method used for MyoMiner. We identified studies that are pertinent to muscle research from GEO and ArrayExpress. Only the studies that provided the raw CEL files proceeded to quality controls. Samples that passed QC were pre-processed with the SCAN algorithm. We thoroughly curated the metadata files and separated them into categories. We used PCA to detect and remove batch effects using the ComBat algorithm. Users have access to muscle tissue and cells gene-pair co-expression, differential co-expression of every category and co-expression networks. All data are available on the MyoMiner web portal.

MyoMiner was constructed in several steps using various tools and processes (Fig. 1). We developed an HTML5 website that allows querying and visualizing for the requested gene correlations. The interface was developed using the Bootstrap responsive framework. Scatterplots and correlation networks are visualized with the Nvd3 and D^3^ [65] JavaScript libraries respectively. All Spearman’s *ρ* and *p*-value pairwise matrices, and metadata are stored on a relational MySQL database which runs under an Apache web server. Dynamic content is processed by the PHP programming language: data retrieval, *ρ* to *Z* transformations and *CI* calculations. The front and back-end is powered by Okeanos [66] cloud services. Complete listings of data series IDs and sample numbers are provided in Additional file 2: Table S7 and Additional file 3: Table S8.

## Utility and discussion

### Data statistics

Following filtering and programmatic retrieval of 81 human (2541 samples) and 198 mouse (2642 samples) muscle series from the ArrayExpress repository, we manually parsed the MIAMI compliant SDRF (sample and data relationship format) metadata file of each series while crosschecking them, if applicable, with the corresponding SOFT (simple omnibus format in text) file from GEO. If there were missing data or differences between ArrayExpress and GEO, we tracked the publication that described the series to correct the missing information. If we still could not extract the missing data, we contacted the corresponding authors in case they could provide us with the correct data. Working in close co-operation with ArrayExpress and GEO personnel, we corrected several series metafiles, although the most common mismatches were copying errors.

We identified and removed 169 human and 144 mouse samples as duplicates. A further 160 human and 122 mouse samples did not pass quality controls and were discarded, leaving us with 2228 human samples (from 74 series) and 2376 mouse samples (from 189 series). The samples were then classified to different categories of 12 or more samples each. In total, 1810 human samples were assigned to 69 categories and 1155 mouse samples were assigned to 73 categories (Table S4).

Categories were created based on gender, age, muscle tissue, condition and strain. A total of 8 skeletal and cardiac muscle tissues are included on MyoMiner together with the combination of those. Human age was classified in years as follows: 0 to 14 as child, 15 to 24 as young, 25 to 59 as adult and 60+ as old. For mouse the classification is in weeks: E (embryonic days) as embryo, 0 to 11 weeks as young, 12 to 24 as adult and 25+ as old. We also included 4 separate strains for mouse: C57BL/6 J, CD1, C3H/HeJ and FVB but also the combinations of them and several other strains (Table 1). The Cells category was derived from mouse microarrays: skeletal muscle precursor cells, cardiomyocytes and immortalized C2C12 mouse cell lines at different stages of differentiation: myoblasts, myotubes 1–2, 3–4 and 5+ days after differentiation. MyoMiner covers 53 distinct conditions including normal and pathological ones. In detail, several exercise categories: aerobic, resistance, endurance, trained or sedentary; different types of diet: high fat or calorie restricted diet; type 2 diabetes (DM2): Pre-DM2, DM2 relatives, etc.; muscle regeneration: cardiotoxin and glycerol injections; several cardiomyopathies: Idiopathic, Dilated, Ischemic and Arrhythmogenic; muscular dystrophies: Duchenne muscular dystrophy, Mdx, Myotonic dystrophy type 2, and many other categories (Additional file 1: Table S4, MyoMiner web portal).

**Table 1:** Gender, age, tissue and strain classification for each organism. Eight distinct muscle tissues, 4 different age stages (years for human and weeks for mouse) and 4 separated mouse strains with their combinations

Organism	Human	Mouse
**Gender**	Both, Male, Female	Both, Male, Female
**Age**	All ages, Child, Young, Adult, Old	All ages, Embryo, Young, Adult, Old
**Anatomic part**	Combined heart, Left ventricle, Both ventricles, Myocardium, Combined skeletal muscle, Quadriceps, Rectus abdominis, Biceps brachii	Combined heart, Left ventricle, Both ventricles, Combined skeletal muscle, Quadriceps, Gastrocnemius, Tibialis anterior, Soleus
**Strain**	NA	Combined, C57BL/6 J, CD1, C3H/HeJ, FVB

To measure the accuracy of the gender prediction method we first tried it on the samples with known gender. For human only 1135 out of 2228 samples had their gender reported. The method classified 98% of the samples to their reported gender. 23 samples (~ 2%) did not match and we investigated further into their original publications. 5 samples out of the 23 were correctly predicted but were reported in the repositories or publications as opposite-sex. This increased the initial match to 98.4%. Regarding the mouse data, the gender was reported in 1390 out of 2376 samples. Again, testing this method on the known gender samples resulted in about a 98% match, with 56 samples being predicted as opposite sex from the ones reported. We identified and corrected 16 falsely reported cases, increasing the prediction match to 98.3% (Additional file 1: Table S5). All gender mismatches that we corrected occurred from copying errors.

### Query results and features

**Fig. 2:**
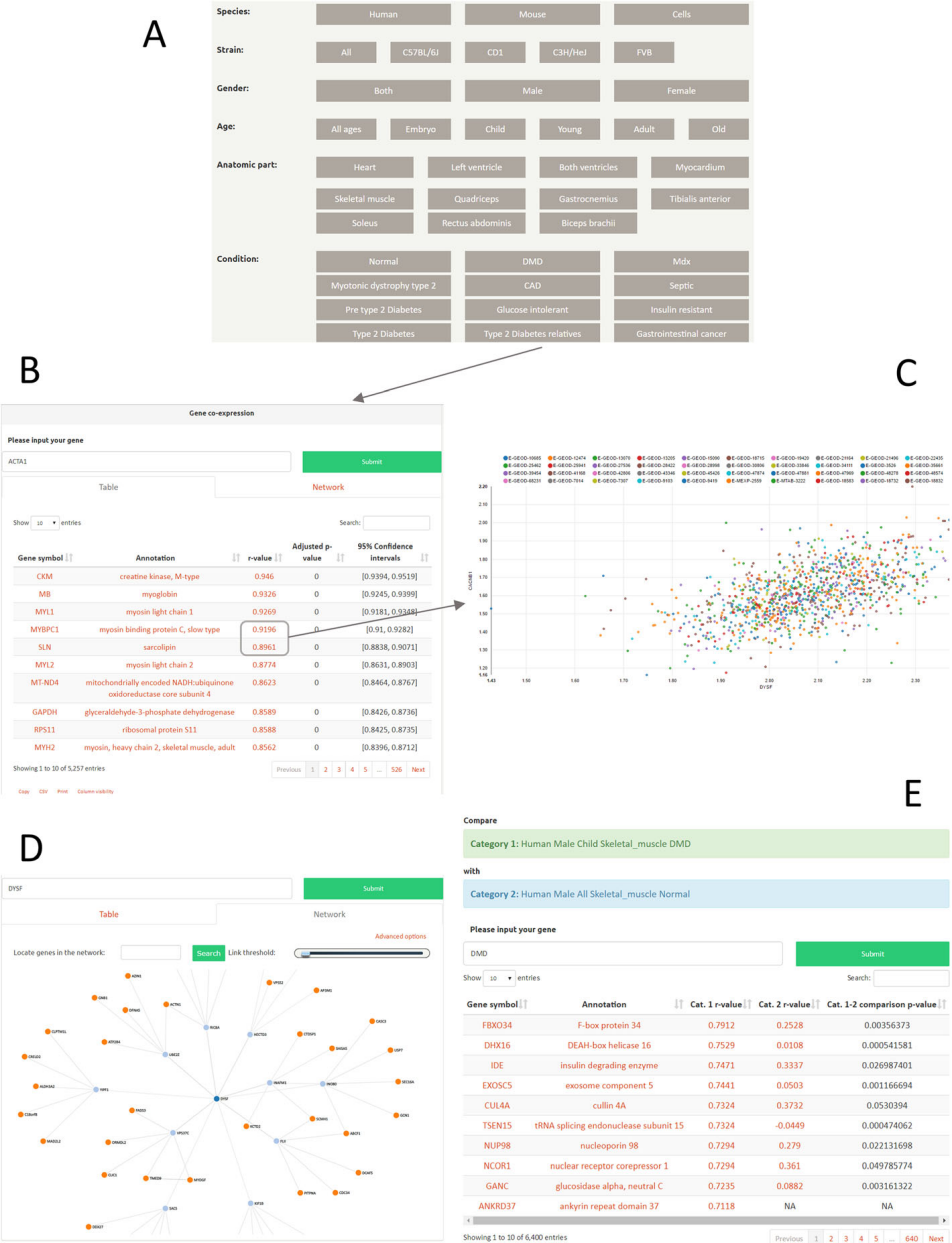
How to browse MyoMiner. **a***Select a category of interest*. All categories are visible at the beginning, so that the user can find with ease what is available on MyoMiner. By clicking a category only the options that are related with this category will remain visible. This way the user is guided to the available MyoMiner categories. **b***Table output*. Search by gene symbol, Ensembl or Entrez gene ID. All transcriptional co-expressions of any expressed gene-pair displayed when hitting submit. The first column is the paired gene symbol, the second is the annotation of the paired gene, the third is the Sprearman’s correlation of that pair, the fourth and fifth are the BH FDR adjusted *p*-value and the confidence intervals. The table can be downloaded in CSV format or copied directly to the clipboard **c***Gene pair scatterplot*. The expression values of every sample of the selected category for that gene pair are plotted by clicking on the r value. Each series is shown at the top and can be toggled to display the expression values for any series independently. **d***Correlation network*. The network is constructed based on gene correlation. Users can change the number of relations or set a correlation threshold from the advanced options. **e***Differential co-expression analysis*. Select two or more categories and compare the first to the rest. A gene may be a regulator if its co-expression is significantly altered (p-value) between pathological conditions. MyoMiner can be accessed at https://www.sys-myo.com/myominer

The MyoMiner interface was designed to enable users to search and quickly retrieve the transcriptional co-expression of any expressed gene pair in muscle tissue and cells. All categories are presented as buttons on the main page (Fig. 2a). When selecting a category, the options that are not relevant to it are deactivated, thereby constraining the search to (and indicating to the user) only those options which remain available. MyoMiner supports queries using HGNC gene symbols (e.g. DYSF), Ensembl IDs (e.g. ENSG00000135636), Entrez gene IDs (e.g. 8291) and Uniprot accession numbers (e.g. O75923). The table output retrieves the correlation values for all expressed gene pairs in the selected category (Fig. **2**b) sorted by *ρ*-value. The first column comprises the paired gene symbols which can also be clicked to search for its list of correlated genes. The second column is a description of the paired gene, also serving as a link to the associated gene on GeneCards [67]. The third column shows the Spearman’s correlation coefficient but also if clicked the scatterplot of this pair. The fourth and fifth columns report two statistic summaries for the user to judge the significance of the correlation: the FDR adjusted *p*-value, and the CI at 95% confidence level, that include information about the estimated effect size and the uncertainty associated with this estimate. CI translates to 95% probability that the population correlation coefficient true *ρ*-value is between *CI*_*lower*_ and *CI*_*upper*_. A search bar is provided on the top right corner of the table format for easy gene pair filtering and the columns can be sorted by clicking on their headers (e.g. sort by positive or negative correlation). The results can be downloaded, in various formats, using the appropriate buttons at the bottom left corner of the table.

Scatterplots are important as supplementary information to help interpret the correlation coefficient. In MyoMiner, interactive expression scatterplots for any gene pair can be accessed by clicking the *ρ*-value. A modal window will appear showing the normalized expression values obtained by SCAN for the selected gene pair (Fig. 2c). The series that were used for the selected category are displayed at the top of the scatterplot. By clicking or double-clicking the series ID, one can either remove the selected series or retain that series only, respectively. Removing series on the scatterplot window will not affect the *ρ*-value as it is pre-computed for all series shown on the scatterplot.

Correlation networks can be accessed by selecting the network tab and pressing the submit button without the need to re-select the category (Fig. 2d). A signed un-weighted 2-shell network will be constructed. It works either with the number of co-expressed genes in each shell (default: 15 and 5 genes for 1st and 2nd shell respectively) or by setting a correlation threshold through the advanced options. A combination of these two methods is also possible.

Another feature is the gene list network, available through the advanced options, where the user can input a list of genes to create the correlation network. In this case, default 1st and 2nd shell values are set to 0 in order to firstly identify if the genes on the list are related. These values can be changed to add co-expressed genes outside from the gene list. The search form “Locate genes in the network” will hide for a short time all the genes in the network except for the searched gene, making it easy to pinpoint the location of genes inside the network. The link threshold bar can be used to remove edges below a certain correlation value, creating sub networks in the process. The blue colored node is used to point the queried gene, the light blue depicts the 1st shell connected nodes and orange the 2nd shell nodes. Users can pan and zoom by click-dragging on an empty space of the interactive network area and using the mouse wheel, respectively. The nodes are interactive and can be moved to any space of the network area. Users can also double-click a node to highlight its immediate connected nodes.

Since correlation networks can grow quite large, including thousands of nodes and many more edges, it could take several minutes to retrieve the values for large networks from the database. For this reason, we decided that network construction will be a client side task, using the D3 JavaScript library. For large networks, we recommend using the Chrome browser as it could take some time to render big networks, especially on low end machines. We also recommend having the graphics card enabled for the browser in order to avoid long rendering time for the network.

Differential co-expression analysis is emerging as a method to complement traditional differential expression analysis [13, 68]. It can detect biologically important differentially co-expressed gene pairs that would otherwise not be detected via co-expression or differential expression [69]. Differentially co-expressed genes between different conditions are likely to be regulators, thus explaining differences between phenotypes [70]. MyoMiner provides differential co-expression analysis for any gene pair from any category combination. In the “Compare gene co-expression” form, users can set the categories for comparison (Fig. **2**e). The first category is compared to the rest after the gene in question is selected. The output includes the gene symbol and its description, the *ρ*_*1*_ value from the first category, the *ρ*_*2*_ value from the second category and the *p*-value of the comparison. If the p-value is higher than 0.05 the difference of *ρ*_*1*_ and *ρ*_*2*_ is not significant at 95% confidence level. MyoMiner supports multiple simultaneous comparisons.

### Improved combined data quality after the correction of batch effects

**Fig. 3:**
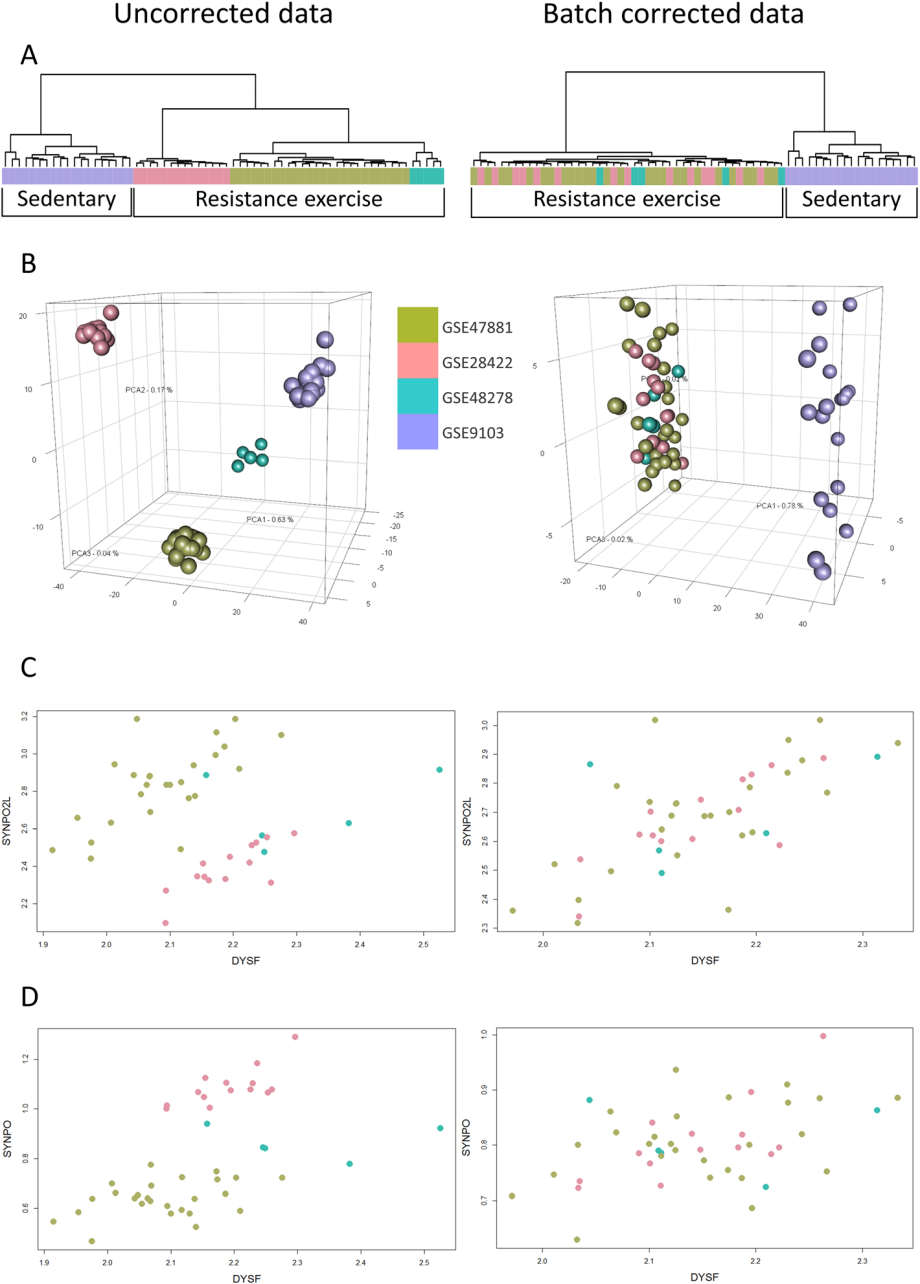
Example of batch effects treatment**.** The adult human quadriceps resistance exercise category is constructed from three series: GSE47881 (olive green), GSE28422 (pink) and GSE48278 (turquoise) that include 45 samples in total. GSE9103 (magenta) series, from sedentary individuals, is used as a visual control. On the left, one can see the untreated samples and on the right the batch-treated samples, using each series as a surrogate. **a** Hierarchical clustering of both resistance exercise and sedentary samples shows a clear separation. Note that resistance exercise samples are clustered by their corresponding series even after pre-processing (normalization). After treating the samples with ComBat, the resistance exercise samples are now mixed, reducing the batch effect. **b** Principal component analysis plots of the same samples. In the untreated plot, samples are clustered very well by their series (olive green, pink and turquoise). However, the resistance exercise series are as far from each other as the sedentary (visual control in this case) series. After the batch correction (right) all resistance exercise samples are clustered together and are clearly separated from the sedentary samples cluster. **c** The expression values of *DYSF* and *SYNPO2L* are grouped by series resulting in a correlation value *r = − 0.05*. After batch correction the samples are mixed with *ρ = 0.62*. **d** Inversely, in the example of *DYSF* and *SYNPO* where the *ρ* value is artificially high, before the treatment (*ρ* = 0.62), the correction reduces it to *ρ* = 0.36

By combining data from different data sets and laboratories from around the world we introduce unwanted technical variation which needs to be corrected. Different processing days between samples in a series were also observed, through PCA plots, as another source of strong non-biological variation [29]. To improve the quality of the co-expression values obtained from tens to hundreds of samples, we check each category for the presence of batch effects by different series and/or processing dates. To acquire the scan dates from the microarray CEL files, we parsed them in text format. We then used PCA to visualize the samples from each category, colored by series or processing dates, on a 3D plane (Fig. 3b), in order to identify underlying batch effects. When we observed non-biological variation we corrected it using the ComBat algorithm [55], as described in the “Batch effects evaluation” section.

Below, we present two examples where batch effect treatment drastically altered the correlation coefficient between the gene pairs (Fig. 3). Dysferlin is a type II transmembrane protein that is enriched in skeletal and cardiac muscle and involved in membrane repair [71]. Mutations or loss of *DYSF* gene lead to muscular dystrophies called dysferlinopathies. Synaptopodin 2-like (*SYNPO2L*) protein is an important paralog of Synaptopodin-2 (*SYNPO2*) that is involved in active binding and bundling and associated with Duchene muscular dystrophy and myofibrilar myopathy 2. We selected the adult human resistance exercise category to illustrate how batch correction removes bias introduced when combining data. Before correction, no strong correlation is observed between *DYSF* and *SYNPO2L*: *ρ = − 0.05* (Table 2, also shown with Pearson’s correlation coefficients).

**Table 2:** Examples of gene pairs correlation changes after batch correction. We illustrate two correlation examples (i) between *DYSF* and *SYNPO2L,* where the correlation increases significantly and (ii) between *DYSF* and *SYNPO,* where the correlation decreases. Both Spearman and Pearson’s correlations are available to indicate that batch effects are prevalent in both parametric and non-parametric statistics. We see considerable changes on their combined correlation coefficients, which is due to the correction of the variation between studies having been done in different labs by different people. In the case of *DYSF* - *SYNPO2L,* originally there seems to be no correlation on the combined samples, despite that a strong positive correlation is observed in each individual series. This bias is removed after batch correction with ComBat, resulting in a positive correlation. The example of the *DYSF* – *SYNPO* pair shows an initial strong positive correlation before batch correction, while the individual series have mixed positive and negative correlations. Following batch correction this value is reduced

	*DYSF* - *SYNPO2L*		*DYSF* - *SYNPO*	
	Spearman *ρ*	Pearson *r*	Spearman *ρ*	Pearson *r*
Uncorrected	−0.05	0.02	0.62	0.53
Batch corrected	0.62	0.65	0.36	0.42
GSE47881	0.6	0.67	0.31	0.39
GSE48278	0.3	0.31	−0.4	−0.08
GSE28422	0.67	0.79	0.64	0.71
Average of the 3 series	0.54	0.62	0.21	0.38

Clustering and PCA plots show that the samples are grouped by series, which may indicate bias (Fig. 3a, b left). The *DYSF* and *SYNPO2L* gene expression scatterplot reveal the extent of the batch effect: even though individual series (different colors) have clear positive correlation the overall correlation is canceled out when combined (Fig. 3c). In detail, the selected category is comprised of three series. Individual series Spearman correlation is GSE47881 *ρ = 0.6*, GSE48278 *ρ = 0.3* and GSE28422 *ρ = 0.67*. We can also average the correlation values using *ρ-*to-*Z* Fisher’s transformation (Additional file 1: Table S6, Eq. S1) to convert the non additive *ρ*-values to *Z* scores, then average the *Z* scores and finally convert the mean *Z* back to *ρ*-value (Additional file 1: Table S6, Eq. S4). *DYSF-SYNPO2L* average *ρ-value* for the category is 0.54. After we treated the samples with ComBat which reduced the aforementioned bias (Fig. 3 a, b, c right) the correlation value increased to 0.62 which could indicate a possible functional association between *DYSF* and *SYNPO2L* [72].

In another example between *DYSF* and Synaptopodin (*SYNPO*), which may be modulating actin-based shape and mobility of dendritic spines, we find that batch effect correction reduces the bias-inflated correlation *ρ = 0.62*. Individual series correlation is as follows: GSE47881 *ρ = 0.31*, GSE48278 *ρ = − 0.4* and GSE28422 *ρ = 0.64.* The scatterplot also reveals that the series have mixed correlations (Fig. 3d left) and the overall *ρ* is biased when we combined the series. The average correlation of the three series is 0.21. After removing the bias (Fig. 3d right) the correlation is reduced from 0.62 to 0.36. Gene pairs that had reduced correlation after batch treatment were more common, suggesting that batch correction reduced the number of false positive correlations.

### Validation of correlation values

We sought to validate the correlation values generated for MyoMiner, by comparison to two existing databases of gene co-expression (MEM and SEEK), and to the GTEx RNA-Seq data compendium. We did this to the extent possible given the limited muscle-specificity and lack of muscle condition sub-categorization of those databases. For this comparison, a panel of 20 muscle-relevant genes (Additional file 4: Table S9) were chosen based on muscle-relevant annotations (Entrez Gene, GeneCards), and on their frequency of representation in consensus lists of the Muscle Gene Sets collection [32]. All 190 pair-wise Pearson correlation values were obtained from MyoMiner for these 20 muscle-relevant genes from MyoMiner’s healthy human whole muscle category (Human|Both genders|All Ages|Skeletal muscle|Normal).

**Fig. 4:**
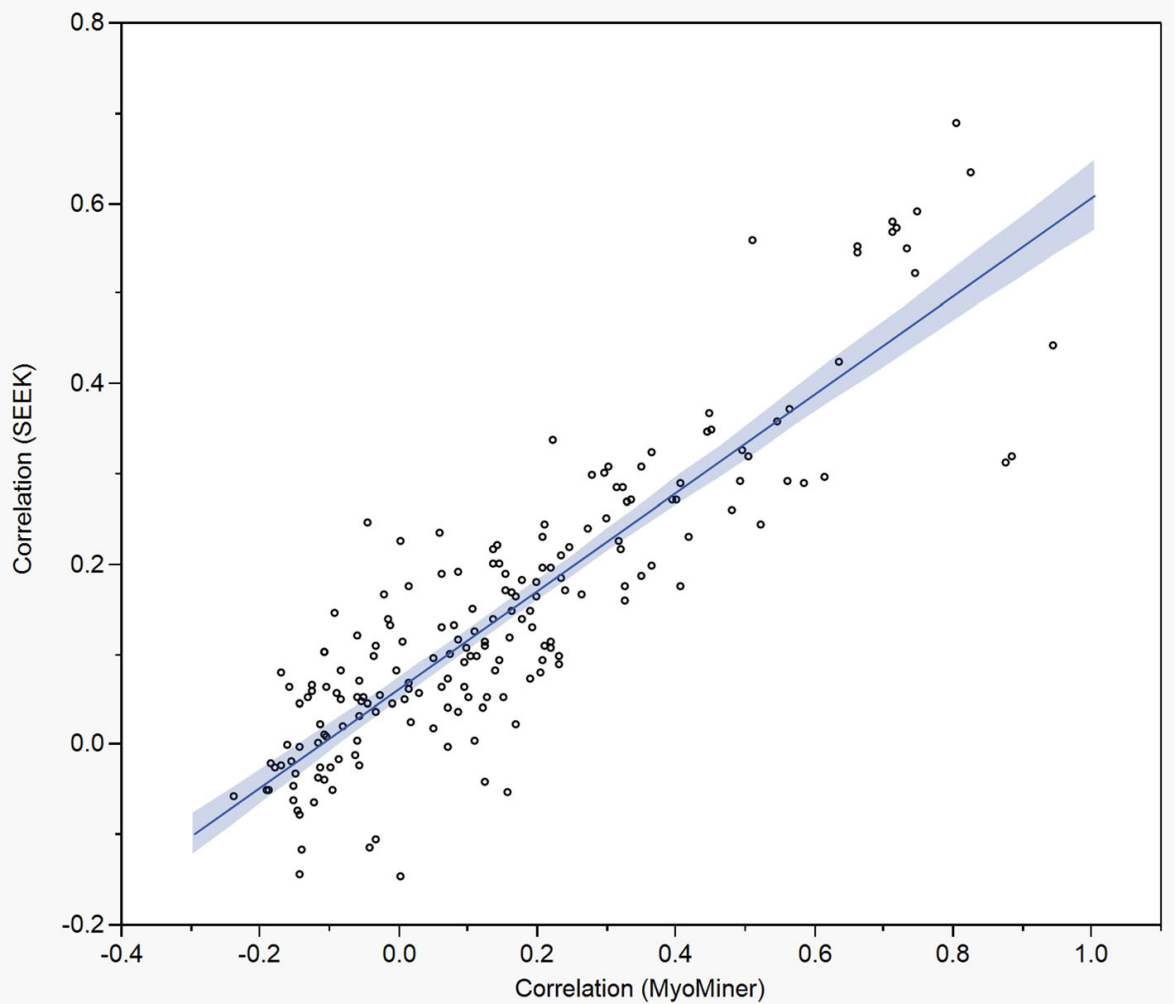
Validation of MyoMiner by comparison to the SEEK co-expression search engine. Pearson correlation values were extracted from both MyoMiner and SEEK for each pair of a panel of 20 muscle-relevant genes (190 pairwise combinations). For MyoMiner, these were obtained for the healthy human whole muscle category (Human|Both genders|All Ages|Skeletal muscle|Normal). For SEEK, the ‘Muscle (Non-cancer)’ dataset was chosen, which combines 87 mostly muscle-related data series from several gene expression platforms. The correlation value from SEEK for a given gene pair was plotted against that from MyoMiner for the same gene pair. The 190 values from MyoMiner correlated with those from SEEK with Pearson *r* of 0.87

The MyoMiner values were compared against similar correlation values for the same pairs of genes given by MEM and SEEK for the closest relevant datasets that we could identify in the MEM and SEEK databases. Pearson correlation could be obtained directly from SEEK, whereas MEM returns a *p*-value for the strength of correlation that is not directly comparable to Pearson, so for MEM we ranked the 190 pair-wise correlations and compared rankings between the two tools. A text search for “Skeletal Muscle” in the MEM tool enabled extraction of correlation values for a dataset that combined 25 muscle-relevant studies on the Affymetrix HG U133 Plus 2.0 array. For SEEK, the ‘Muscle (Non-cancer)’ dataset was chosen, which combines 87 mostly muscle-related data series from several gene expression platforms. We observed strong agreement in correlation values for MyoMiner with SEEK (Pearson *r* = 0.87; Fig. 4) and with MEM (Spearman *ρ* = 0.74; Additional file 1: Fig. S1).

Correlation values for healthy whole muscle were calculated from GTEx RNA-Seq data in a similar way to those calculated for MyoMiner’s healthy human whole muscle category. A reasonable level of agreement was observed in correlation values between MyoMiner and GTEx (Pearson *r* = 0.66; Additional file 1: Fig. S1).

## Conclusions

In this work we retrieved and analyzed striated muscle pertinent microarray samples and combined them effectively for the construction of a muscle-tissue-specific co-expression database. MyoMiner provides a simple, effective and easy way to identify co-expressed gene pairs under a vast number of experimental conditions. This was not available in any existing co-expression database. Thus, MyoMiner represents a powerful tool for muscle researchers, helping them to delineate gene function and key regulators.

For MyoMiner we chose to use the Spearman correlation coefficient, despite the fact that Pearson correlation seems to be more popular in other correlation databases. We did not use the Pearson correlation because it is sensitive to outliers and because of the assumptions that need to be met, in order to calculate adjusted *p*-values: every gene would have to be normally distributed, while gene pairs have to be bivariately normally distributed. On the other hand, Spearman correlation is robust to outliers and does not require assumptions of linearity. To determine the strength of the correlation we have provided the adjusted p-value and the confidence intervals.

It is noteworthy that the most correlated partners for a driver gene may vary significantly between co-expression databases. This could be attributed to different transcriptomic platforms, although most of these databases use GPL570 and GPL1261 platforms as we did. Moreover, different pre-processing methods, batch effect correction methods or the lack thereof, tissue- and cell-specific expression, variable cell states, different correlation coefficients, and other factors, add to the differences found between co-expression databases. An investigation of the inconsistencies between co-expression databases could identify common gene characteristics or the key factors that contribute to those differences. Our comparison of MyoMiner to the MEM and SEEK databases suggests that there is reasonable agreement of these resources in terms of co-expressed gene pairs in healthy muscle, validating the approach used in MyoMiner, and supporting the trustworthiness of MyoMiner’s correlation values for muscle diseases and other muscle conditions.

One caveat of gene expression correlation is that it can be driven by other factors. For example, a transcription factor (TF), when upregulated, drives the expression of gene X and Y. In this scenario, TF with X and TF with Y will be highly correlated. However, X and Y will be highly correlated as well, since both are upregulated from the same TF. This could be beneficial as X and Y could be involved in the same processes, but if we are interested specifically in the relation of X with Y, their correlation would be zero if TF was not upregulated. In order to extract the correlation between X and Y without TF interfering, we can calculate the partial correlation [73]. Partial correlation could theoretically be used to remove all the gene effects from a pair of genes, but it would require more microarray experiments than the number of genes. It has been used successfully to create relatively small networks [74].

To derive statistical confidence across large numbers of experiments, and because we wished MyoMiner to examine co-expression differences between experimental conditions (some of which have low quantities of published expression data), we have focused our analysis on the most-used microarray platforms for human and murine muscle studies. Microarrays have been extremely useful in a wide area of biological applications, but they also have a number of limitations. Importantly, a microarray can only detect RNA sequences that the designed probes can detect. Simply put, if the RNA contains sequences that have no corresponding oligos in the array, the sequences will not be measured. In gene expression analysis, a gene that was not described before will not be present in the array. Also, non-coding RNA sequences are typically not present on arrays. This problem is more pronounced in older arrays where only a set number of probes could be printed on the array; thus a portion of the genes could eventually be measured. Newer commercial arrays have tried to compensate for this by including probes that do not match to any known genes at the time they are designed - predicted transcripts which can then be assigned to newly discovered genes if their sequences match. Also, as time progresses more researchers are using the now popular BrainArray CDF repository which is updated annually. Another difficulty in terms of probe design, is to generate probes of which the RNA sequences do not overlap. If sequences are homologous, then a probe could detect multiple genes at once, which is particularly problematic for genes with many splice variants or for genes that belong to the same family. Dai et al. [40], address this issue by selecting probes that detect specific and unique parts of the gene (whenever this is possible). It should be noted that specific arrays can detect splice variants by having probes which detect specific exons or exon junctions [75–77]. Moreover, microarrays measure, by design, relative concentration indirectly. The intensity measured in a probe is proportional to the concentration of a sequence that can hybridize to this probe. However, experimental spike-in studies [78] showed that the probe intensity is nonlinearly proportional to the target concentration [79–81]. The array will become saturated at high target concentrations, while at low concentrations there will be no binding. The intensities are linear within a very limited range of RNA concentration. Another limitation is that co-expression analyses based on a single microarray platform may have technical biases associated with that platform: for example, the properties of cross-hybridization and the dynamic range of probes differ among microarray platforms, as does the signal-to-noise ratio [18]. These limitations of microarray technology may to some extent account for the stronger agreement of MyoMiner’s correlation values with those of tools such SEEK (*r* = 0.88) and MEM (*ρ* = 0.74), which use mainly microarray data, compared with the moderate agreement with correlation values obtained from the GTEx RNA-Seq dataset (*r* = 0.66). For these reasons, it may be useful in future work to cross-reference common co-expression patterns found in MyoMiner against those found in RNA-Seq data in order to identify whether any consistent disparities are present that may be resulting from technical biases in the microarray chip – such instances could then be filtered out of MyoMiner findings for specific experimental categories. Published RNA-Seq datasets are becoming more numerous in the neuromuscular field but remain limited in number especially when considering specific experimental or pathological conditions.

A major reason for creating MyoMiner is to be able to compare gene co-expression networks between conditions – i.e. to identify cases were the co-expression of a given gene pair is lost, gained, or inverted, as a result of, for example, a pathological genetic mutation or an environmental change. For individual gene pairs, this is currently facilitated by the MyoMiner web interface, and the MyoMiner database itself opens up the possibility of a systematic analysis in the future. Clearly, such analysis is not possible without measuring co-expression separately in the conditions that are to be compared. However, a caveat of this approach is that a highly specific phenotype might lack biological variation to an extent that gene co-expression becomes overly noisy. Our comparisons to healthy skeletal muscle sub-sets of the SEEK and MEM databases suggest that there is consistency of identified co-expressed gene pairs for this biological condition, indicating that noise does not dominate, and boding well for other conditions. However, care should be taken and high confidence *p*-values should be sought when comparing between very precise biological conditions in MyoMiner, especially for murine samples, in which inter-individual variation could be relatively minor.

Finally, it could be interesting in future iterations of MyoMiner to examine the relationship of gene co-expression with other types of gene and protein functional association (e.g. [82–84]) especially for the identification of functional modules [85].

The MyoMiner database is a powerful tool for muscle researchers to investigate gene function, based on tissue specific co-expression, and new disease mechanics, based on changes in co-expression between normal and pathological tissues.

## Supplementary information

**Additional file 1.** **Figure S1.** Pair-wise correlations of 20 selected muscle genes in MyoMiner compared to the same pairwise correlations in GTEx and MEM, for healthy human whole muscle tissue. **Table S1.** Alternative IDs to the originals A-AFFY-44 for human UG-U133 Plus 2.0 and A-AFFY-45 for mouse MG 430 2 arrays. **Table S2** - **Table S3.** Failed quality samples and series from the human and mouse microarray data collection. **Table S4.** Number of samples, series and expressed genes for each of 69 and 73 categories in human and mouse respectively. **Table S5.** Samples with opposite gender prediction. **Table S6.** Supplementary equations for ρ to Ζ and Z to ρ transformations, and confidence intervals calculation.

**Additional file 2.** **Table S7.** Complete listing of data series IDs for human experiments.

**Additional file 3.** **Table S8.** Complete listing of data series IDs for murine experiments.

**Additional file 4.** **Table S9.** The panel of 20 muscle-relevant genes used for validation of MyoMiner co-expression values. This panel is based on muscle-relevant annotations, and frequency of representation in consensus lists of the Muscle Gene Sets collection.

## Data Availability

The transcriptomic data that support the findings of this study are available from ArrayExpress, https://www.ebi.ac.uk/arrayexpress/, and the Gene Expression Omnibus, https://www.ncbi.nlm.nih.gov/geo/. Complete listings of data series IDs and sample numbers are provided in Additional file 2: Table S7 and Additional file 3: Table S8. All the data generated for MyoMiner are available at https://sys-myo.com/myominer/.

## Abbreviations

GEO: Gene Expression Omnibus
FDR: False discovery rate
CDF: Chip description file
MIAME: Minimum Information About a Microarray Experiment
CEL: The file format of the Affymetrix raw (light intensity) file
RLE: Relative Log Expression
NUSE: Normalized Unscaled Standard Error
SCAN: Single Channel Array Normalization
RMA: Robust Multi-array Average
GC-RMA: GeneChip Robust Multi-array Average
UPC: Universal exPression Code
HGNC: HUGO Gene Nomenclature Committee
DM2: Diabetes Mellitus type 2
PCA: Principal Component Analysis
TF: Transcription Factor
