# Supplementary material for: MyoMiner: explore gene co-expression in normal and pathological muscle
Source: BMC Med Genomics. 2020 May 11;13:67. doi: 10.1186/s12920-020-0712-3 (PMC7216615; doi:10.1186/s12920-020-0712-3)
Supplement: Supplementary file 1 — Additional file 1. Figure S1. Pair-wise correlations of 20 selected muscle genes in MyoMiner compared to the same pairwise correlations in GTEx and MEM, for healthy human whole muscle tissue. Table S1. Alternative IDs to the originals A-AFFY-44 for human UG-U133 Plus 2.0 and A-AFFY-45 for mouse MG 430 2 arrays. Table S2 - Table S3. Failed quality samples and series from the human and mouse microarray data collection. Table S4. Number of samples, series and expressed genes for each of 69 and 73 categories in human and mouse respectively. Table S5. Samples with opposite gender prediction. Table S6. Supplementary equations for ρ to Ζ and Z to ρ transformations, and confidence intervals calculation. [file 12920_2020_712_MOESM1_ESM.docx]

**A**
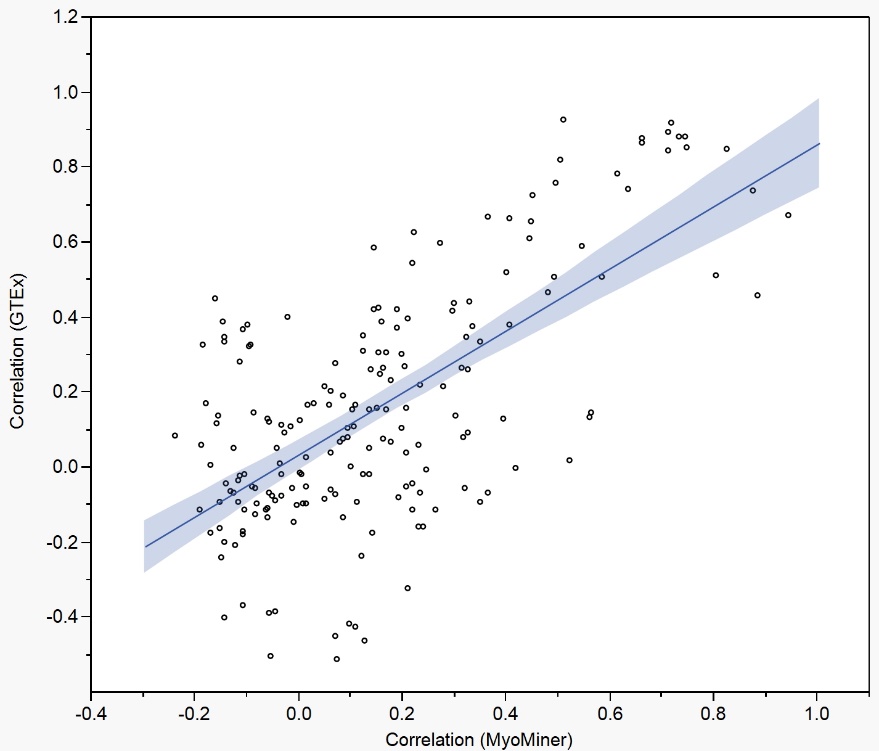


**B
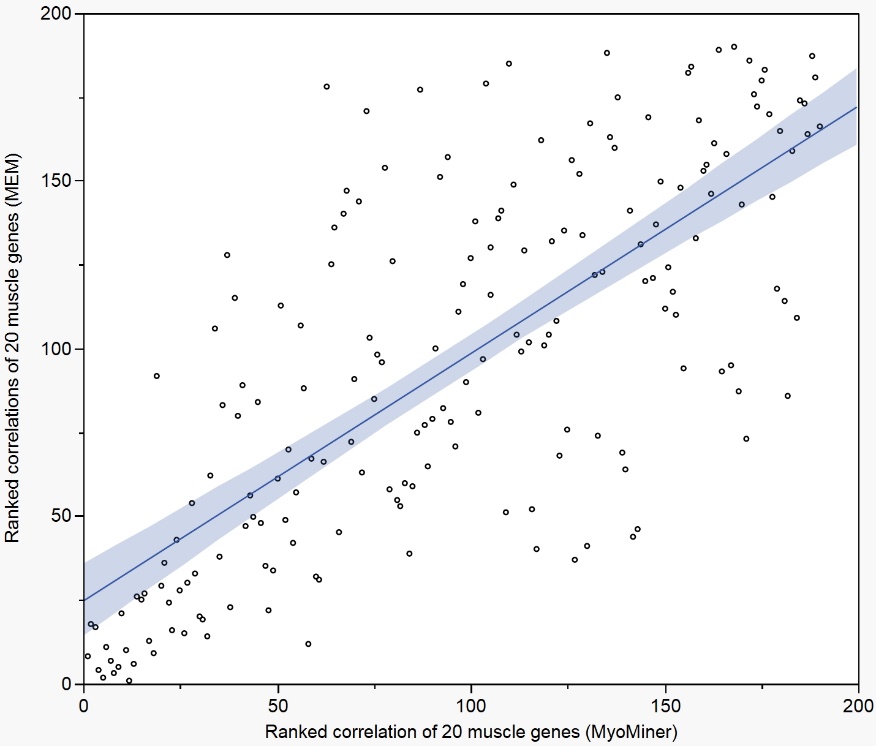
**

**Figure S1 |** Pair-wise correlations of 20 selected muscle genes in MyoMiner compared to the same pairwise correlations in GTEx and MEM, for healthy human whole muscle tissue. **S1a.** Direct comparison of Spearman correlation values *ρ* in MyoMiner (x-axis) compared to those of GTEx (y-axis). Overall Pearson’s r correlation of MyoMiner with GTEx was 0.66. **S1b.** Comparison of rankings of correlations in MyoMiner (x-axis) with those of MEM (y-axis). Correlation values for MyoMiner are calculated as Spearman *ρ*, while those of MEM are calculated as p-values that indicating the strength of co-expression (lower values equal stronger relationship). Overall Spearman *ρ* correlation of the rank values for MyoMiner with MEM was 0.74. Lines of best fit and their confidence intervals are indicated.

**Table S1 |** Alternative IDs to the originals A-AFFY-44 for human UG-U133 Plus 2.0 and A-AFFY-45 for mouse MG 430 2 arrays. These experiments get an alternative ID even if they use the same chip because they mapped the probes to probesets and then to transcripts or genes with a different Chip Description File (CDF) than the original. For the muscle microarray collection, we pinpointed and downloaded three more series for human and two for mouse.

| Affymetrix GeneChip Human Genome U133 Plus 2.0 alternative ArrayExpress IDs | Affymetrix GeneChip Mouse Genome 430 2.0 alternative ArrayExpress IDs |
| --- | --- |
| \| A-GEOD-4454 \| A-GEOD-10184 \| A-GEOD-16268 \| \| --- \| --- \| --- \| \| A-GEOD-4866 \| A-GEOD-10274 \| A-GEOD-16273 \| \| A-GEOD-5760 \| A-GEOD-10335 \| A-GEOD-16311 \| \| A-GEOD-6671 \| A-GEOD-10371 \| A-GEOD-16356 \| \| A-GEOD-6732 \| A-GEOD-10881 \| A-GEOD-16372 \| \| A-GEOD-6791 \| A-GEOD-10925 \| A-GEOD-17175 \| \| A-GEOD-6823 \| A-GEOD-11084 \| A-GEOD-17180 \| \| A-GEOD-6879 \| A-GEOD-11364 \| A-GEOD-17392 \| \| A-GEOD-7566 \| A-GEOD-11433 \| A-GEOD-17394 \| \| A-GEOD-7567 \| A-GEOD-11670 \| A-GEOD-17810 \| \| A-GEOD-7869 \| A-GEOD-13232 \| A-GEOD-17811 \| \| A-GEOD-8019 \| A-GEOD-13668 \| A-GEOD-17929 \| \| A-GEOD-8542 \| A-GEOD-13695 \| A-GEOD-17996 \| \| A-GEOD-8715 \| A-GEOD-13916 \| A-GEOD-18121 \| \| A-GEOD-9099 \| A-GEOD-14837 \| A-GEOD-18478 \| \| A-GEOD-9101 \| A-GEOD-14877 \| A-GEOD-18850 \| \| A-GEOD-9102 \| A-GEOD-15308 \| A-GEOD-19109 \| \| A-GEOD-9419 \| A-GEOD-15394 \| A-GEOD-19171 \| \| A-GEOD-9454 \| A-GEOD-15445 \| A-GEOD-19883 \| \| A-GEOD-9486 \| A-GEOD-15676 \| A-GEOD-19918 \| \| A-GEOD-9987 \| A-GEOD-16006 \| A-GEOD-20182 \| \| A-GEOD-10175 \| A-GEOD-16100 \| A-MEXP-2335 \| | \| A-GEOD-5008 \| A-GEOD-14657 \| \| --- \| --- \| \| A-GEOD-5759 \| A-GEOD-14661 \| \| A-GEOD-5766 \| A-GEOD-14757 \| \| A-GEOD-6456 \| A-GEOD-14996 \| \| A-GEOD-6526 \| A-GEOD-15041 \| \| A-GEOD-6886 \| A-GEOD-15592 \| \| A-GEOD-7368 \| A-GEOD-15722 \| \| A-GEOD-7546 \| A-GEOD-15967 \| \| A-GEOD-7635 \| A-GEOD-16225 \| \| A-GEOD-8059 \| A-GEOD-16368 \| \| A-GEOD-8462 \| A-GEOD-16582 \| \| A-GEOD-8492 \| A-GEOD-17109 \| \| A-GEOD-9523 \| A-GEOD-17114 \| \| A-GEOD-9746 \| A-GEOD-18078 \| \| A-GEOD-10288 \| A-GEOD-18122 \| \| A-GEOD-10369 \| A-GEOD-18223 \| \| A-GEOD-10773 \| A-GEOD-18376 \| \| A-GEOD-11044 \| A-GEOD-18416 \| \| A-GEOD-13502 \| A-GEOD-18615 \| \| A-GEOD-13621 \| A-GEOD-18854 \| \| A-GEOD-13763 \| A-GEOD-20766 \| |

Table S2 | Samples and series removed from the human microarray data collection that failed to pass quality controls. In total 160 samples were considered of low quality and were not used for any further analyses. All samples are removed from the gray shaded series.

| \| Series \| Sample \| Reason \| \| --- \| --- \| --- \| \| E-GEOD-1145 \| GSM18435_PA-D_93_2.CEL \| NUSE above 1.1 and RLE wider than +-0.2 \| \| E-GEOD-12486 \| GSM313633.CEL \| NUSE above 1.1 and RLE wider than +-0.2 \| \| E-GEOD-13070 \| GSM342678.CEL \| Low percent present compared to other samples from the same series. Also, NUSE above 1.1 and RLE wider than +-0.2 \| \| E-GEOD-13070 \| GSM342677.CEL \| Low percent present compared to other samples from the same series. Also, NUSE above 1.1 and RLE wider than +-0.2 \| \| E-GEOD-13070 \| GSM342673.CEL \| Low percent present compared to other samples from the same series. Also, NUSE above 1.1 and RLE wider than +-0.2 \| \| E-GEOD-13070 \| GSM342808.CEL \| Low percent present compared to other samples from the same series. Also, NUSE above 1.1 and RLE wider than +-0.2 \| \| E-GEOD-13070 \| GSM342814.CEL \| Low percent present compared to other samples from the same series. Also, NUSE above 1.1 and RLE wider than +-0.2 \| \| E-GEOD-13070 \| GSM342821.CEL \| Low percent present compared to other samples from the same series. Also, NUSE above 1.1 and RLE wider than +-0.2 \| \| E-GEOD-13070 \| GSM342836.CEL \| Low percent present compared to other samples from the same series. Also, NUSE above 1.1 and RLE wider than +-0.2 \| \| E-GEOD-13070 \| GSM342850.CEL \| Low percent present compared to other samples from the same series. Also, NUSE above 1.1 and RLE wider than +-0.2 \| \| E-GEOD-13070 \| GSM342857.CEL \| Low percent present compared to other samples from the same series. Also, NUSE above 1.1 and RLE wider than +-0.2 \| \| E-GEOD-13070 \| GSM342879.CEL \| Low percent present compared to other samples from the same series. Also, NUSE above 1.1 and RLE wider than +-0.2 \| \| E-GEOD-13070 \| GSM342884.CEL \| Low percent present compared to other samples from the same series. Also, NUSE above 1.1 and RLE wider than +-0.2 \| \| E-GEOD-13070 \| GSM342888.CEL \| Low percent present compared to other samples from the same series. Also, NUSE above 1.1 and RLE wider than +-0.2 \| \| E-GEOD-13070 \| GSM342900.CEL \| Low percent present compared to other samples from the same series. Also, NUSE above 1.1 and RLE wider than +-0.2 \| \| E-GEOD-13070 \| GSM342931.CEL \| Low percent present compared to other samples from the same series. Also, NUSE above 1.1 and RLE wider than +-0.2 \| \| E-GEOD-13205 \| GSM333440.CEL \| Low percent present compared to other samples from the same series. Also, NUSE above 1.1 and RLE wider than +-0.2 \| \| E-GEOD-15090 \| GSM377469.CEL \| Low percent present compared to other samples from the same series. Also, NUSE above 1.1 and RLE wider than +-0.2 \| \| E-GEOD-18715 \| GSM464627_C12.CEL \| RLE wider than +-0.2 \| \| E-GEOD-19420 \| GSM482956.CEL \| RLE wider than +-0.2 \| \| E-GEOD-22435 \| GSM557526.CEL \| Low percent present compared to other samples from the same series. Also, NUSE above 1.1 and RLE wider than +-0.2 \| \| E-GEOD-24199 \| 8 samples \| Whole series due to Low percent present and other fluctuations \| \| E-GEOD-24235 \| GSM596038.CEL \| Low percent present compared to other samples from the same series \| \| E-GEOD-24235 \| GSM595901.CEL \| Low percent present compared to other samples from the same series \| \| E-GEOD-25462 \| GSM624971.CEL \| Low percent present compared to other samples from the same series. Also, NUSE above 1.1 and RLE wider than +-0.2 \| \| E-GEOD-25462 \| GSM624970.CEL \| Low percent present compared to other samples from the same series. Also, NUSE above 1.1 and RLE wider than +-0.2 \| \| E-GEOD-25462 \| GSM624938.CEL \| Low percent present compared to other samples from the same series. Also, NUSE above 1.1 and RLE wider than +-0.2 \| \| E-GEOD-28392 \| 70 samples \| Whole series due to actin3/actin5 ration being 3 times higher than the recommended limits and RLE widen than +-0.2 \| \| E-GEOD-28422 \| GSM702359.CEL \| RLE wider than +-0.2 \| \| E-GEOD-28422 \| GSM702374.CEL \| RLE wider than +-0.2 \| \| E-GEOD-28422 \| GSM702438.CEL \| RLE wider than +-0.2 \| \| E-GEOD-28422 \| GSM702442.CEL \| RLE wider than +-0.2 \| \| E-GEOD-34111 \| GSM842037.CEL \| Low percent present compared to other samples from the same series. Also, NUSE above 1.1 and RLE wider than +-0.2. Also actin3/5 and gapdh3/5 higher than recommended values \| \| E-GEOD-34111 \| GSM842028.CEL \| Low percent present compared to other samples from the same series. Also, NUSE above 1.1 and RLE wider than +-0.2. Also actin3/5 and gapdh3/5 higher than recommended values \| \| E-GEOD-34111 \| GSM842024.CEL \| Low percent present compared to other samples from the same series. Also, NUSE above 1.1 and RLE wider than +-0.2. Also actin3/5 and gapdh3/5 higher than recommended values \| \| E-GEOD-34111 \| GSM842022.CEL \| Low percent present compared to other samples from the same series. Also, NUSE above 1.1 and RLE wider than +-0.2. Also actin3/5 and gapdh3/5 higher than recommended values \| \| E-GEOD-34111 \| GSM842018.CEL \| Low percent present compared to other samples from the same series. Also, NUSE above 1.1 and RLE wider than +-0.2. Also actin3/5 and gapdh3/5 higher than recommended values \| \| E-GEOD-34111 \| GSM842017.CEL \| Low percent present compared to other samples from the same series. Also, NUSE above 1.1 and RLE wider than +-0.2. Also actin3/5 and gapdh3/5 higher than recommended values \| \| E-GEOD-34111 \| GSM842014.CEL \| Low percent present compared to other samples from the same series. Also, NUSE above 1.1 and RLE wider than +-0.2. Also actin3/5 and gapdh3/5 higher than recommended values \| \| E-GEOD-3526 \| GSM80797.CEL \| Low percent present compared to other samples from the same series. RLE wider than +-0.2 \| \| E-GEOD-3526 \| GSM80796.CEL \| Low percent present compared to other samples from the same series. RLE wider than +-0.2 \| \| E-GEOD-38780 \| GSM949395_AA12_15_11_D8.CEL \| RLE wider than +-0.2 \| \| E-GEOD-39454 \| GSM969502_MA45_GEIM385.CEL \| RLE wider than +-0.2 \| \| E-GEOD-39454 \| GSM969496_MA45_GEIM375.CEL \| RLE wider than +-0.2 \| \| E-GEOD-39454 \| GSM969489_MA45_GEIM354.CEL \| RLE wider than +-0.2 \| \| E-GEOD-40231 \| GSM988933_STAGE_9_SKLM.CEL \| Low percent present compared to other samples from the same series. Also, NUSE above 1.1 and RLE wider than +-0.2 \| \| E-GEOD-40231 \| GSM988889_STAGE_59_SKLM.CEL \| Low percent present compared to other samples from the same series. Also, NUSE above 1.1 and RLE wider than +-0.2 \| \| E-GEOD-40231 \| GSM988877_STAGE_56_SKLM.CEL \| Low percent present compared to other samples from the same series. Also, NUSE above 1.1 and RLE wider than +-0.2 \| \| E-GEOD-40231 \| GSM988762_STAGE_31_SKLM.CEL \| Low percent present compared to other samples from the same series. Also, NUSE above 1.1 and RLE wider than +-0.2 \| \| E-GEOD-40231 \| GSM988759_STAGE_30_SKLM.CEL \| Low percent present compared to other samples from the same series. Also, NUSE above 1.1 and RLE wider than +-0.2 \| \| E-GEOD-45426 \| GSM1104107_S26.CEL \| RLE wider than +-0.2 \| \| E-GEOD-45426 \| GSM1104095_S14.CEL \| RLE wider than +-0.2 \| \| E-GEOD-47874 \| GSM1161401_75_51545Pre.CEL \| RLE wider than +-0.2 \| \| E-GEOD-47881 \| GSM1161775_D4_BH073F.CEL \| Low percent present compared to other samples from the same series. Also, NUSE above 1.1 and RLE wider than +-0.4 \| \| E-GEOD-47881 \| GSM1161834_D79_PC035F.CEL \| Low percent present compared to other samples from the same series. Also, NUSE above 1.1 and RLE wider than +-0.4 \| \| E-GEOD-47969 \| GSM1163791_DUKE38_334.CEL \| Low percent present compared to other samples from the same series. Also, NUSE above 1.1 and RLE wider than +-0.4 \| \| E-GEOD-48278 \| GSM1174154_MJH_STRRIDE_S401_F_POST_1_HG-U133_Plus_2_.CEL \| Low percent present compared to other samples from the same series. Also, NUSE above 1.1 and RLE wider than +-0.2 \| \| E-GEOD-48278 \| GSM1174122_MJH_STRRIDE_S317_F_POST_1_HG-U133_Plus_2_.CEL \| Low percent present compared to other samples from the same series. Also, NUSE above 1.1 and RLE wider than +-0.2 \| \| E-GEOD-48278 \| GSM1174123_MJH_STRRIDE_S317_F_PRE_1_HG-U133_Plus_2_.CEL \| Low percent present compared to other samples from the same series. Also, NUSE above 1.1 and RLE wider than +-0.2 \| \| E-GEOD-48278 \| GSM1174116_MJH_STRRIDE_S301_E_POST_1_HG-U133_Plus_2_.CEL \| Low percent present compared to other samples from the same series. Also, NUSE above 1.1 and RLE wider than +-0.2 \| \| E-GEOD-48278 \| GSM1174096_MJH_STRRIDE_S235_C_POST_1_HG-U133_Plus_2_.CEL \| Low percent present compared to other samples from the same series. Also, NUSE above 1.1 and RLE wider than +-0.2 \| \| E-GEOD-48278 \| GSM1174061_MJH_STRRIDE_S172_A_PRE_1_HG-U133_Plus_2_.CEL \| Low percent present compared to other samples from the same series. Also, NUSE above 1.1 and RLE wider than +-0.2 \| \| E-GEOD-48278 \| GSM1174053_MJH_STRRIDE_S156_A_PRE_1_HG-U133_Plus_2_.CEL \| Low percent present compared to other samples from the same series. Also, NUSE above 1.1 and RLE wider than +-0.2 \| \| E-GEOD-48278 \| GSM1174050_MJH_STRRIDE_S146_C_POST_1_HG-U133_Plus_2_.CEL \| Low percent present compared to other samples from the same series. Also, NUSE above 1.1 and RLE wider than +-0.2 \| \| E-GEOD-48278 \| GSM1174106_MJH_STRRIDE_S257_D_POST_1_HG-U133_Plus_2_.CEL \| Low percent present compared to other samples from the same series. Also, NUSE above 1.1 and RLE wider than +-0.2 \| \| E-GEOD-62203 \| 8 samples \| Whole series due to Low percent present and other fluctuations \| \| E-GEOD-7014 \| GSM161970.CEL \| Low percent present compared to other samples from the same series. Also, NUSE above 1.1 and RLE wider than +-0.2 \| \| E-GEOD-7014 \| GSM161944.CEL \| Low percent present compared to other samples from the same series. Also, NUSE above 1.1 and RLE wider than +-0.2 \| \| E-GEOD-7014 \| GSM161943.CEL \| Low percent present compared to other samples from the same series. Also, NUSE above 1.1 and RLE wider than +-0.2 \| \| E-MTAB-37 \| A-673_SS271874_HG-U133_Plus_2_HCHP-167937_.CEL \| RLE wider than +-0.2 \| \| E-MTAB-37 \| RD_SS275763_HG-U133_Plus_2_HCHP-170309_.CEL \| RLE wider than +-0.2 \| \| E-GEOD-18732 \| GSM465386.CEL \| Low percent present compared to other samples from the same series. Also, NUSE above 1.1 and RLE wider than +-0.2 \| \| E-GEOD-18732 \| GSM465281.CEL \| Low percent present compared to other samples from the same series. Also, NUSE above 1.1 and RLE wider than +-0.2 \| \| E-GEOD-18732 \| GSM465319.CEL \| Low percent present compared to other samples from the same series. Also, NUSE above 1.1 and RLE wider than +-0.2 \| \| E-GEOD-9103 \| GSM230397.cel \| RLE wider than +-0.2 \| \| E-GEOD-9103 \| GSM230407.cel \| RLE wider than +-0.3 \| \| E-GEOD-9103 \| GSM230418.cel \| RLE wider than +-0.4 \| |
| --- | --- | --- | --- | --- | --- | --- | --- | --- | --- | --- | --- | --- | --- | --- | --- | --- | --- | --- | --- | --- | --- | --- | --- | --- | --- | --- | --- | --- | --- | --- | --- | --- | --- | --- | --- | --- | --- | --- | --- | --- | --- | --- | --- | --- | --- | --- | --- | --- | --- | --- | --- | --- | --- | --- | --- | --- | --- | --- | --- | --- | --- | --- | --- | --- | --- | --- | --- | --- | --- | --- | --- | --- | --- | --- | --- | --- | --- | --- | --- | --- | --- | --- | --- | --- | --- | --- | --- | --- | --- | --- | --- | --- | --- | --- | --- | --- | --- | --- | --- | --- | --- | --- | --- | --- | --- | --- | --- | --- | --- | --- | --- | --- | --- | --- | --- | --- | --- | --- | --- | --- | --- | --- | --- | --- | --- | --- | --- | --- | --- | --- | --- | --- | --- | --- | --- | --- | --- | --- | --- | --- | --- | --- | --- | --- | --- | --- | --- | --- | --- | --- | --- | --- | --- | --- | --- | --- | --- | --- | --- | --- | --- | --- | --- | --- | --- | --- | --- | --- | --- | --- | --- | --- | --- | --- | --- | --- | --- | --- | --- | --- | --- | --- | --- | --- | --- | --- | --- | --- | --- | --- | --- | --- | --- | --- | --- | --- | --- | --- | --- | --- | --- | --- | --- | --- | --- | --- | --- | --- | --- | --- | --- | --- | --- | --- | --- | --- | --- | --- | --- | --- | --- | --- | --- | --- | --- | --- | --- | --- | --- | --- | --- | --- | --- | --- |

Table S3 | Samples and series removed from the mouse microarray data collection that failed to pass quality controls. In total 122 samples were considered of low quality and were not used for any further analyses. All samples are removed from the gray shaded series.

| \| **Series** \| **Samples** \| **Reason** \| \| --- \| --- \| --- \| \| E-GEOD-12730 \| GSM319343.CEL \| Low percent present compared to other samples from the same series. Also, NUSE above 1.1 and RLE wider than +-0.2 \| \| E-GEOD-13347 \| GSM313205.CEL \| Low percent present compared to other samples from the same series. Also, NUSE above 1.1 and RLE wider than +-0.2 \| \| E-GEOD-16438 \| GSM413181.CEL \| Low percent present compared to other samples from the same series. Also, NUSE above 1.1 and RLE wider than +-0.2 \| \| E-GEOD-16438 \| GSM413176.CEL \| Low percent present compared to other samples from the same series. Also, NUSE above 1.1 and RLE wider than +-0.2 \| \| E-GEOD-16438 \| GSM413161.CEL \| NUSE above 1.1 and RLE wider than +-0.2 \| \| E-GEOD-16486 \| GSM414370.CEL \| Low percent present compared to other samples from the same series. Also, NUSE above 1.1 and RLE wider than +-0.2 \| \| E-GEOD-18033 \| 56 samples \| Whole series due to abnormal high percent present \| \| E-GEOD-25908 \| GSM636278.cel \| NUSE above 1.1 and RLE wider than +-0.2 \| \| E-GEOD-25908 \| GSM636225.cel \| NUSE above 1.1 and RLE wider than +-0.2 \| \| E-GEOD-38870 \| 18 samples \| Whole series due to Low percent present and other fluctuations \| \| E-GEOD-43373 \| GSM1061639_Mus_SE2__D4_13515.CEL \| Low percent present compared to other samples from the same series. Also, NUSE above 1.1 and RLE wider than +-0.2 \| \| E-GEOD-43373 \| GSM1061638_Mus_SE2__D4_13514.CEL \| Low percent present compared to other samples from the same series. Also, NUSE above 1.1 and RLE wider than +-0.2 \| \| E-GEOD-43779 \| GSM1071181_Rahme_04-06-10_2-AA_treated_muscle_4D_replicate_3.CEL \| Low percent present compared to other samples from the same series. Also, NUSE above 1.1 and RLE wider than +-0.2 \| \| E-GEOD-43779 \| GSM1071179_Rahme_04-06-10_2-AA_treated_muscle_4D_replicate_1.CEL \| Low percent present compared to other samples from the same series. Also, NUSE above 1.1 and RLE wider than +-0.2 \| \| E-GEOD-45577 \| GSM1109982_NUID-0000-0150-3235.cel \| RLE off limits \| \| E-GEOD-45577 \| GSM1109962_NUID-0000-0150-3224.cel \| RLE off limits \| \| E-GEOD-45577 \| GSM1109961_NUID-0000-0150-3205.cel \| RLE off limits \| \| E-GEOD-45577 \| GSM1109960_NUID-0000-0150-3200.cel \| RLE off limits \| \| E-GEOD-45577 \| GSM1109959_NUID-0000-0150-3238.cel \| RLE off limits \| \| E-GEOD-47104 \| GSM1144810_KT-13MB6D2F1old_5.14.08_2.CEL \| Low percent present compared to other samples from the same series. Also, NUSE above 1.1 and RLE wider than +-0.2 \| \| E-GEOD-47104 \| GSM1144808_KT-11MB6D2F1adult_5.14.08_2.CEL \| Low percent present compared to other samples from the same series. Also, NUSE above 1.1 and RLE wider than +-0.2 \| \| E-GEOD-6398 \| GSM147516.CEL \| Low percent present compared to other samples from the same series. Also, NUSE above 1.1 and RLE wider than +-0.2 \| \| E-GEOD-65927 \| GSM1611277_15_4semN1.CEL \| Low percent present compared to other samples from the same series. Also, NUSE above 1.1 and RLE wider than +-0.2 \| \| E-GEOD-65927 \| GSM1611276_14_4semB.CEL \| Low percent present compared to other samples from the same series. Also, NUSE above 1.1 and RLE wider than +-0.2 \| \| E-GEOD-65927 \| GSM1611275_13_4semA.CEL \| Low percent present compared to other samples from the same series. Also, NUSE above 1.1 and RLE wider than +-0.2 \| \| E-GEOD-7605 \| GSM183976.CEL \| NUSE above 1.1 and RLE wider than +-0.2 \| \| E-GEOD-7605 \| GSM183977.CEL \| NUSE above 1.1 and RLE wider than +-0.2 \| \| E-MEXP-1623 \| C9.CEL \| Low percent present compared to other samples from the same series. Also, NUSE above 1.1 and RLE wider than +-0.2 \| \| E-MEXP-2446 \| 681WTmuscleLADROSEMOU_03_080814.CEL \| Low percent present compared to other samples from the same series. Also, NUSE above 1.1 and RLE wider than +-0.2 \| \| E-MEXP-2446 \| 482WTmuscleShamROSEMOU_02_080814.CEL \| Low percent present compared to other samples from the same series. Also, NUSE above 1.1 and RLE wider than +-0.2 \| \| E-GEOD-12337 \| GSM309962.CEL \| Low percent present compared to other samples from the same series. Also, NUSE above 1.1 and RLE wider than +-0.2 \| \| E-GEOD-13031 \| GSM326496.cel \| NUSE above 1.1 and RLE wider than +-0.2 \| \| E-GEOD-13874 \| GSM349106.CEL \| NUSE above 1.1 and RLE wider than +-0.2 \| \| E-GEOD-13874 \| GSM349107.CEL \| NUSE above 1.1 and RLE wider than +-0.2 \| \| E-GEOD-13874 \| GSM349108.CEL \| NUSE above 1.1 and RLE wider than +-0.2 \| \| E-GEOD-1479 \| GSM25168.CEL \| NUSE above 1.1 and RLE wider than +-0.2 \| \| E-GEOD-19079 \| GSM472351.CEL \| Low percent present compared to other samples from the same series. Also, NUSE above 1.1 and RLE wider than +-0.2 \| \| E-GEOD-21368 \| GSM372908.CEL \| Low percent present compared to other samples from the same series. Also, NUSE above 1.1 and RLE wider than +-0.2 \| \| E-GEOD-23101 \| GSM569342.CEL \| NUSE above 1.1 and RLE wider than +-0.2 \| \| E-GEOD-23101 \| GSM569339.CEL \| NUSE above 1.1 and RLE wider than +-0.2 \| \| E-GEOD-30164 \| 4 samples \| Very low percent present 1.4-4% \| \| E-GEOD-50399 \| GSM1218142_3wks_cko3.CEL \| NUSE above 1.1 and RLE wider than +-0.2 \| \| E-GEOD-5500 \| GSM126911.CEL \| Low percent present compared to other samples from the same series. Also, NUSE above 1.1 and RLE wider than +-0.2 \| \| E-GEOD-62049 \| GSM1518961_CD117310DN_Mouse430_2_.CEL \| RLE off limits \| \| E-GEOD-7424 \| GSM179576.CEL \| NUSE above 1.1 and RLE wider than +-0.2 \| \| E-GEOD-8199 \| GSM202774.CEL \| Low percent present compared to other samples from the same series. Also, NUSE above 1.1 and RLE wider than +-0.2 \| \| E-GEOD-58676 \| GSM1416750_GFP_S48_3.CEL \| Low percent present compared to other samples from the same series. Also, NUSE above 1.1 and RLE wider than +-0.2 \| |
| --- | --- | --- | --- | --- | --- | --- | --- | --- | --- | --- | --- | --- | --- | --- | --- | --- | --- | --- | --- | --- | --- | --- | --- | --- | --- | --- | --- | --- | --- | --- | --- | --- | --- | --- | --- | --- | --- | --- | --- | --- | --- | --- | --- | --- | --- | --- | --- | --- | --- | --- | --- | --- | --- | --- | --- | --- | --- | --- | --- | --- | --- | --- | --- | --- | --- | --- | --- | --- | --- | --- | --- | --- | --- | --- | --- | --- | --- | --- | --- | --- | --- | --- | --- | --- | --- | --- | --- | --- | --- | --- | --- | --- | --- | --- | --- | --- | --- | --- | --- | --- | --- | --- | --- | --- | --- | --- | --- | --- | --- | --- | --- | --- | --- | --- | --- | --- | --- | --- | --- | --- | --- | --- | --- | --- | --- | --- | --- | --- | --- | --- | --- | --- | --- | --- | --- | --- | --- | --- | --- | --- | --- | --- | --- | --- |

Table S4 | Number of samples, series and expressed genes for each of 69 and 73 categories in human and mouse respectively. The last column indicates which of the categories were corrected for batch effects.

| \| **Organism** \| **Category (Anatomic part, condition, gender, age, strain mouse specific)** \| **Samples** \| **Series** \| **Expressed genes** \| **Batch corrected** \| \| --- \| --- \| --- \| --- \| --- \| --- \| \| **Human** \| Heart, Normal, Both, All \| 60 \| 10 \| 5280 \| Yes \| \|  \| Heart, Normal, Male, All \| 43 \| 9 \| 4934 \| Yes \| \|  \| Heart, Normal, Female, All \| 17 \| 6 \| 5879 \| Yes \| \|  \| Heart, Normal, Both, Old \| 15 \| 4 \| 4289 \| Yes \| \|  \| Heart, Normal, Both, Adult \| 20 \| 5 \| 4535 \| Yes \| \|  \| Heart - Myocardium, Normal, Both, All \| 19 \| 4 \| 3773 \| No \| \|  \| Heart - Left ventricle, Normal, Both, All \| 31 \| 6 \| 6108 \| Yes \| \|  \| Heart - Left ventricle, Normal, Female, All \| 12 \| 5 \| 6397 \| Yes \| \|  \| Heart- Left ventricle, Normal, Male, All \| 19 \| 5 \| 5873 \| Yes \| \|  \| Heart - Both ventricles, Normal, Both, All \| 37 \| 6 \| 5899 \| Yes \| \|  \| Heart - Left ventricle, Idiopathic cardiomyopathy, Both, All \| 26 \| 1 \| 6184 \| Yes \| \|  \| Heart - Left ventricle, Idiopathic cardiomyopathy, Male, All \| 16 \| 1 \| 6319 \| Yes \| \|  \| Heart - Loth ventricles, Arrhythmogenic right ventricular cardiomyopathy, Both, All \| 12 \| 1 \| 3880 \| Yes \| \|  \| Heart, Dilated cardiomyopathy, Both, All \| 35 \| 2 \| 4451 \| Yes \| \|  \| Heart - Myocardium, Dilated cardiomyopathy, Both, Adult \| 21 \| 1 \| 3809 \| Yes \| \|  \| Heart - Both ventricles, Dilated cardiomyopathy, Both, All \| 14 \| 1 \| 5111 \| Yes \| \|  \| Heart, Ischemic cardiomyopathy, Both, All \| 55 \| 6 \| 6536 \| Yes \| \|  \| Skeletal muscle - Rectus abdominis, Upper gastrointestinal cancer, Both, All \| 17 \| 1 \| 4035 \| No \| \|  \| Skeletal muscle - Quadriceps, Type 2 diabetes DM2, Both, All \| 68 \| 4 \| 3738 \| Yes \| \|  \| Skeletal muscle - Quadriceps, Type 2 diabetes DM2, Female, Adult \| 16 \| 2 \| 3268 \| Yes \| \|  \| Skeletal muscle - Quadriceps, Type 2 diabetes DM2, Male, All \| 52 \| 4 \| 3913 \| Yes \| \|  \| Skeletal muscle - Quadriceps, Thiazolidinedione TZD PPAR gamma ligand treatment for 3 months, All, All \| 16 \| 1 \| 3722 \| No \| \|  \| Skeletal muscle - Quadriceps, Septic, All, All \| 12 \| 1 \| 4741 \| No \| \|  \| Skeletal muscle - Quadriceps, Pre type 2 diabetes DM2, Male, All \| 12 \| 1 \| 5653 \| Yes \| \|  \| Skeletal muscle - Quadriceps, Post hyperinsulinemic euglycemic clamp, All, All \| 16 \| 1 \| 4000 \| No \| \|  \| Skeletal muscle - Quadriceps, Post hyperinsulinemic euglycemic clamp thiazolidinedione TZD PPAR gamma ligand treatment for 3 months, All, All \| 17 \| 1 \| 4065 \| No \| \|  \| Skeletal muscle, Myotonic dystrophy type 2, All, All \| 20 \| 1 \| 3863 \| No \| \|  \| Skeletal muscle - Quadriceps, Insulin resistant polycystic ovary syndrome PCOS, Female, Adult \| 16 \| 1 \| 2828 \| No \| \|  \| Skeletal muscle - Quadriceps, Insulin resistant, All, Adult \| 38 \| 1 \| 3724 \| No \| \|  \| Skeletal muscle - Quadriceps, Insulin resistant thiazolidinedione TZD PPAR gamma ligand treatment for 3 months, All, Adult \| 46 \| 1 \| 3721 \| No \| \|  \| Skeletal muscle - Quadriceps, Insulin resistant post hyperinsulinemic euglycemic clamp, All, Adult \| 42 \| 1 \| 3955 \| No \| \|  \| Skeletal muscle - Quadriceps, Insulin resistant post hyperinsulinemic euglycemic clamp thiazolidinedione TZD no response to treatment, All, Adult \| 12 \| 1 \| 3582 \| No \| \|  \| Skeletal muscle - Quadriceps, Insulin resistant post hyperinsulinemic euglycemic clamp thiazolidinedione TZD response to treatment, All, Adult \| 12 \| 1 \| 3631 \| No \| \|  \| Skeletal muscle - Quadriceps, Insulin resistant post hyperinsulinemic euglycemic clamp thiazolidinedione TZD unresponsive to treatment, All, Adult \| 25 \| 1 \| 3556 \| No \| \|  \| Skeletal muscle - Quadriceps, Glucose intolerant, All, All \| 26 \| 1 \| 3408 \| No \| \|  \| Skeletal muscle - Rectus abdominis, Coronary Artery Disease, All, All \| 61 \| 1 \| 4339 \| No \| \|  \| Skeletal muscle - Quadriceps, Chronic Obstructive Pulmonary disease sedentary, All, Old \| 15 \| 1 \| 4580 \| Yes \| \|  \| Skeletal muscle - Quadriceps, Chronic Obstructive Pulmonary disease trained, All, old \| 15 \| 1 \| 4407 \| No \| \|  \| Skeletal muscle - Quadriceps, Chronic Obstructive Pulmonary disease, All, Old \| 30 \| 1 \| 4509 \| No \| \|  \| Skeletal muscle - Quadriceps, Calorie restrictive for 12 weeks, Female, All \| 14 \| 1 \| 3484 \| No \| \|  \| Skeletal muscle, Normal, All, All \| 1107 \| 46 \| 5257 \| Yes \| \|  \| Skeletal muscle, Normal, Female, All \| 438 \| 33 \| 5524 \| Yes \| \|  \| Skeletal muscle, Normal, Male, All \| 666 \| 41 \| 5103 \| Yes \| \|  \| Skeletal muscle - Biceps brachii, Normal, All, All \| 45 \| 5 \| 3984 \| Yes \| \|  \| Skeletal muscle - Quadriceps, Normal, All, All \| 994 \| 32 \| 5298 \| Yes \| \|  \| Skeletal muscle - Rectus abdominis, Normal, All, All \| 13 \| 2 \| 4050 \| Yes \| \|  \| Skeletal muscle - Quadriceps, Normal, Female, All \| 379 \| 20 \| 5631 \| Yes \| \|  \| Skeletal muscle - Quadriceps, Normal, Male, All \| 614 \| 28 \| 5125 \| Yes \| \|  \| Skeletal muscle, Normal, All, young \| 192 \| 17 \| 4071 \| Yes \| \|  \| Skeletal muscle, Normal, All, adult \| 565 \| 25 \| 5298 \| Yes \| \|  \| Skeletal muscle, Normal, All, old \| 261 \| 21 \| 7020 \| Yes \| \|  \| Skeletal muscle - Quadriceps, First degree diabetes relative, All, All \| 39 \| 2 \| 3656 \| Yes \| \|  \| Skeletal muscle - Quadriceps, Reported protein intake 0.75 g kg, Male, All \| 22 \| 1 \| 3115 \| No \| \|  \| Skeletal muscle - Quadriceps, Reported protein intake 0.75 g kg, Male, adult \| 12 \| 1 \| 3154 \| No \| \|  \| Skeletal muscle - Quadriceps, Reported protein intake 1.00 g kg, Male, All \| 22 \| 1 \| 3018 \| No \| \|  \| Skeletal muscle - Quadriceps, Reported protein intake 1.00 g kg, Male, adult \| 12 \| 1 \| 3059 \| No \| \|  \| Skeletal muscle - Quadriceps, Reported protein intake 0.50 g kg, Male, All \| 22 \| 1 \| 3178 \| No \| \|  \| Skeletal muscle - Quadriceps, Reported protein intake 0.50 g kg, Male, adult \| 12 \| 1 \| 3188 \| No \| \|  \| Skeletal muscle, Resistance exercise, All, All \| 114 \| 6 \| 6167 \| Yes \| \|  \| Skeletal muscle, Resistance exercise, Female, All \| 42 \| 6 \| 7154 \| Yes \| \|  \| Skeletal muscle, Resistance exercise, Male, All \| 73 \| 6 \| 5827 \| Yes \| \|  \| Skeletal muscle, Resistance exercise, All, Young \| 39 \| 4 \| 4319 \| Yes \| \|  \| Skeletal muscle - Quadriceps, Resistance exercise, All, Adult \| 45 \| 3 \| 7172 \| Yes \| \|  \| Skeletal muscle - Quadriceps, Resistance exercise, All, Old \| 30 \| 3 \| 7817 \| Yes \| \|  \| Skeletal muscle - Quadriceps, Trained, All, All \| 38 \| 2 \| 7915 \| Yes \| \|  \| Skeletal muscle - Quadriceps, Endurance exercise, All, All \| 42 \| 2 \| 3170 \| Yes \| \|  \| Skeletal muscle - Quadriceps, Aerobic exercise, All, All \| 27 \| 1 \| 5297 \| No \| \|  \| Skeletal muscle - Quadriceps, Sedentary, All, All \| 38 \| 3 \| 3493 \| Yes \| \|  \| Skeletal muscle, DMD, Male, Child \| 16 \| 1 \| 6400 \| Yes \| \| **Mouse** \| Heart, Normal, Both, All, All \| 296 \| 65 \| 5437 \| Yes \| \|  \| Heart, Normal, Male, All, All \| 219 \| 49 \| 5371 \| Yes \| \|  \| Heart, Normal, Female, All, All \| 63 \| 19 \| 5479 \| Yes \| \|  \| Heart, Normal, Both, Young, All \| 161 \| 38 \| 5763 \| Yes \| \|  \| Heart, Normal, Male, Young, All \| 107 \| 26 \| 5765 \| Yes \| \|  \| Heart, Normal, Female, Young, All \| 44 \| 13 \| 5578 \| Yes \| \|  \| Heart, Normal, Both, Adult, All \| 80 \| 16 \| 4834 \| Yes \| \|  \| Heart, Normal, Both, Old, All \| 32 \| 9 \| 5508 \| Yes \| \|  \| Heart, Normal, Male, Young, C3H-HeJ \| 16 \| 1 \| 6140 \| Yes \| \|  \| Heart - Cardiomyocyte, Normal, Both, All, All \| 18 \| 5 \| 6178 \| Yes \| \|  \| Heart - Left ventricle, Normal, Both, All, All \| 38 \| 9 \| 5302 \| Yes \| \|  \| Heart - Both ventricles, Normal, Both, All, All \| 55 \| 12 \| 5569 \| Yes \| \|  \| Heart, Normal, Both, All, CD1 \| 28 \| 4 \| 6359 \| Yes \| \|  \| Heart, Normal, Both, All, C57BL-6J \| 140 \| 33 \| 5318 \| Yes \| \|  \| Heart, Normal, Male, All, C57BL-6J \| 105 \| 27 \| 5183 \| Yes \| \|  \| Heart, Normal, Female, All, C57BL-6J \| 26 \| 6 \| 5441 \| Yes \| \|  \| Heart, Normal, Both, Young, C57BL-6J \| 76 \| 18 \| 5261 \| Yes \| \|  \| Heart, Normal, Female, Young, C57BL-6J \| 23 \| 5 \| 5380 \| Yes \| \|  \| Heart, Normal, Male, Young, C57BL-6J \| 47 \| 12 \| 4829 \| Yes \| \|  \| Heart, Normal, All, Adult, C57BL-6J \| 36 \| 9 \| 4802 \| Yes \| \|  \| Heart, Normal, Male, Old, C57BL-6J \| 16 \| 5 \| 5493 \| Yes \| \|  \| Heart, Normal, Both, Embryo, All \| 88 \| 10 \| 7318 \| Yes \| \|  \| Heart, Aortic banding, Both, All, All \| 14 \| 3 \| 6538 \| Yes \| \|  \| Heart, Calorie restricted diet, Both, All, All \| 15 \| 3 \| 4529 \| Yes \| \|  \| Heart, Sham, Both, All, All \| 23 \| 4 \| 5566 \| Yes \| \|  \| Heart, Transverse aortic constriction, Both, All, All \| 14 \| 2 \| 5765 \| Yes \| \|  \| Skeletal muscle - Precursor cells, Normal, Male, All, All \| 14 \| 1 \| 4125 \| Yes \| \|  \| Skeletal muscle - Gastrocnemius, Tenotomy, Male, Young, C57BL-6J \| 17 \| 1 \| 4439 \| No \| \|  \| Skeletal muscle - Gastrocnemius, Sham, Both, Young, All \| 17 \| 2 \| 4638 \| Yes \| \|  \| Skeletal muscle, Sham, Male, All, All \| 13 \| 2 \| 5552 \| Yes \| \|  \| Skeletal muscle, Calpain3 knockout, Male, All, All \| 21 \| 1 \| 4745 \| No \| \|  \| Skeletal muscle - Tibialis anterior, Cardiotoxin injection, Male, Adult, C57BL-6J \| 20 \| 1 \| 8245 \| No \| \|  \| Skeletal muscle - Gastrocnemius, Casting, Male, Young, C57BL-6J \| 25 \| 1 \| 5885 \| No \| \|  \| Skeletal muscle - Tibialis anterior, Glycerol injection, Male, Adult, C57BL-6J \| 18 \| 1 \| 8672 \| No \| \|  \| Skeletal muscle, Mdx, Male, Young, All \| 16 \| 4 \| 6445 \| Yes \| \|  \| Skeletal muscle, High fat diet, Both, All, All \| 99 \| 6 \| 4727 \| Yes \| \|  \| Skeletal muscle, High fat diet, Male, Young, All \| 16 \| 2 \| 4692 \| Yes \| \|  \| Skeletal muscle, High fat diet, Both, Adult, All \| 83 \| 6 \| 4714 \| Yes \| \|  \| Skeletal muscle - Gastocnemius, High fat diet, Both, Adult, C57BL-6J \| 15 \| 2 \| 4603 \| Yes \| \|  \| Skeletal muscle - Quadriceps, High fat diet, Male, Adult, C57BL-6J \| 22 \| 2 \| 4614 \| Yes \| \|  \| Skeletal muscle, Normal, Both, All, All \| 346 \| 57 \| 5216 \| Yes \| \|  \| Skeletal muscle, Normal, Female, All, All \| 44 \| 17 \| 4860 \| Yes \| \|  \| Skeletal muscle, Normal, Female, Adult, All \| 14 \| 4 \| 4786 \| Yes \| \|  \| Skeletal muscle, Normal, Female, Young, All \| 15 \| 7 \| 5270 \| Yes \| \|  \| Skeletal muscle - Quadriceps, Normal, Female, All, All \| 15 \| 5 \| 4747 \| Yes \| \|  \| Skeletal muscle, Normal, Both, Old, All \| 42 \| 8 \| 5117 \| Yes \| \|  \| Skeletal muscle - Gastrocnemius, Normal, Male, Old, All \| 18 \| 4 \| 5803 \| Yes \| \|  \| Skeletal muscle - Quadriceps, Normal, Male, Old, All \| 14 \| 3 \| 4591 \| Yes \| \|  \| Skeletal muscle, Normal, Both, Adult, All \| 119 \| 19 \| 5033 \| Yes \| \|  \| Skeletal muscle, Normal, Both, Adult, C57BL-6J \| 59 \| 8 \| 4884 \| Yes \| \|  \| Skeletal muscle, Normal, Male, Adult, FVB \| 15 \| 2 \| 5128 \| Yes \| \|  \| Skeletal muscle - Gastrocnemius, Normal, Both, Adult, All \| 32 \| 6 \| 4535 \| Yes \| \|  \| Skeletal muscle - Quadriceps, Normal, Both, Adult, All \| 49 \| 6 \| 4996 \| Yes \| \|  \| Skeletal muscle, Normal, Both, Young, All \| 145 \| 26 \| 5381 \| Yes \| \|  \| Skeletal muscle, Normal, Both, Young, C57BL-6J \| 79 \| 11 \| 5473 \| Yes \| \|  \| Skeletal muscle - Quadriceps, Normal, Both, Young, All \| 15 \| 5 \| 4492 \| Yes \| \|  \| Skeletal muscle, Normal, Male, All, All \| 305 \| 46 \| 5294 \| Yes \| \|  \| Skeletal muscle - Gastrocnemius, Normal, Both, All, All \| 127 \| 16 \| 5473 \| Yes \| \|  \| Skeletal muscle - Quadriceps, Normal, Both, All, All \| 81 \| 14 \| 4892 \| Yes \| \|  \| Skeletal muscle - Soleus, Normal, Both, All, All \| 22 \| 3 \| 4884 \| Yes \| \|  \| Skeletal muscle - Tibialis anterior, Normal, Both, All, All \| 15 \| 5 \| 6971 \| Yes \| \|  \| Skeletal muscle, Normal, Male, Old, All \| 34 \| 6 \| 5172 \| Yes \| \|  \| Skeletal muscle, Normal, Male, Adult, All \| 105 \| 15 \| 5056 \| Yes \| \|  \| Skeletal muscle, Normal, Male, Adult, C57BL-6J \| 52 \| 6 \| 4930 \| Yes \| \|  \| Skeletal muscle - Gastrocnemius, Normal, Male, Adult, All \| 23 \| 4 \| 4646 \| Yes \| \|  \| Skeletal muscle - Quadriceps, Normal, Male, Adult, All \| 45 \| 5 \| 4925 \| Yes \| \|  \| Skeletal muscle, Normal, Male, Young, All \| 127 \| 21 \| 5403 \| Yes \| \|  \| Skeletal muscle, Normal, Male, Young, C57BL-6J \| 72 \| 8 \| 5506 \| Yes \| \|  \| Skeletal muscle - Gastrocnemius, Normal, Male, Young, All \| 76 \| 8 \| 5665 \| Yes \| \|  \| Skeletal muscle - C2C12, Normal undifferentiated, Female, All, All \| 20 \| 6 \| 7121 \| Yes \| \|  \| Skeletal muscle - C2C12, Normal 1-2 days differentiated, Female, All, All \| 39 \| 2 \| 5815 \| Yes \| \|  \| Skeletal muscle - C2C12, Normal 3-4 days differentiated, Female, All, All \| 14 \| 5 \| 6999 \| Yes \| \|  \| Skeletal muscle - C2C12, Normal 5 days differentiated, Female, All, All \| 12 \| 4 \| 7366 \| Yes \| |
| --- | --- | --- | --- | --- | --- | --- | --- | --- | --- | --- | --- | --- | --- | --- | --- | --- | --- | --- | --- | --- | --- | --- | --- | --- | --- | --- | --- | --- | --- | --- | --- | --- | --- | --- | --- | --- | --- | --- | --- | --- | --- | --- | --- | --- | --- | --- | --- | --- | --- | --- | --- | --- | --- | --- | --- | --- | --- | --- | --- | --- | --- | --- | --- | --- | --- | --- | --- | --- | --- | --- | --- | --- | --- | --- | --- | --- | --- | --- | --- | --- | --- | --- | --- | --- | --- | --- | --- | --- | --- | --- | --- | --- | --- | --- | --- | --- | --- | --- | --- | --- | --- | --- | --- | --- | --- | --- | --- | --- | --- | --- | --- | --- | --- | --- | --- | --- | --- | --- | --- | --- | --- | --- | --- | --- | --- | --- | --- | --- | --- | --- | --- | --- | --- | --- | --- | --- | --- | --- | --- | --- | --- | --- | --- | --- | --- | --- | --- | --- | --- | --- | --- | --- | --- | --- | --- | --- | --- | --- | --- | --- | --- | --- | --- | --- | --- | --- | --- | --- | --- | --- | --- | --- | --- | --- | --- | --- | --- | --- | --- | --- | --- | --- | --- | --- | --- | --- | --- | --- | --- | --- | --- | --- | --- | --- | --- | --- | --- | --- | --- | --- | --- | --- | --- | --- | --- | --- | --- | --- | --- | --- | --- | --- | --- | --- | --- | --- | --- | --- | --- | --- | --- | --- | --- | --- | --- | --- | --- | --- | --- | --- | --- | --- | --- | --- | --- | --- | --- | --- | --- | --- | --- | --- | --- | --- | --- | --- | --- | --- | --- | --- | --- | --- | --- | --- | --- | --- | --- | --- | --- | --- | --- | --- | --- | --- | --- | --- | --- | --- | --- | --- | --- | --- | --- | --- | --- | --- | --- | --- | --- | --- | --- | --- | --- | --- | --- | --- | --- | --- | --- | --- | --- | --- | --- | --- | --- | --- | --- | --- | --- | --- | --- | --- | --- | --- | --- | --- | --- | --- | --- | --- | --- | --- | --- | --- | --- | --- | --- | --- | --- | --- | --- | --- | --- | --- | --- | --- | --- | --- | --- | --- | --- | --- | --- | --- | --- | --- | --- | --- | --- | --- | --- | --- | --- | --- | --- | --- | --- | --- | --- | --- | --- | --- | --- | --- | --- | --- | --- | --- | --- | --- | --- | --- | --- | --- | --- | --- | --- | --- | --- | --- | --- | --- | --- | --- | --- | --- | --- | --- | --- | --- | --- | --- | --- | --- | --- | --- | --- | --- | --- | --- | --- | --- | --- | --- | --- | --- | --- | --- | --- | --- | --- | --- | --- | --- | --- | --- | --- | --- | --- | --- | --- | --- | --- | --- | --- | --- | --- | --- | --- | --- | --- | --- | --- | --- | --- | --- | --- | --- | --- | --- | --- | --- | --- | --- | --- | --- | --- | --- | --- | --- | --- | --- | --- | --- | --- | --- | --- | --- | --- | --- | --- | --- | --- | --- | --- | --- | --- | --- | --- | --- | --- | --- | --- | --- | --- | --- | --- | --- | --- | --- | --- | --- | --- | --- | --- | --- | --- | --- | --- | --- | --- | --- | --- | --- | --- | --- | --- | --- | --- | --- | --- | --- | --- | --- | --- | --- | --- | --- | --- | --- | --- | --- | --- | --- | --- | --- | --- | --- | --- | --- | --- | --- | --- | --- | --- | --- | --- | --- | --- | --- | --- | --- | --- | --- | --- | --- | --- | --- | --- | --- | --- | --- | --- | --- | --- | --- | --- | --- | --- | --- | --- | --- | --- | --- | --- | --- | --- | --- | --- | --- | --- | --- | --- | --- | --- | --- | --- | --- | --- | --- | --- | --- | --- | --- | --- | --- | --- | --- | --- | --- | --- | --- | --- | --- | --- | --- | --- | --- | --- | --- | --- | --- | --- | --- | --- | --- | --- | --- | --- | --- | --- | --- | --- | --- | --- | --- | --- | --- | --- | --- | --- | --- | --- | --- | --- | --- | --- | --- | --- | --- | --- | --- | --- | --- | --- | --- | --- | --- | --- | --- | --- | --- | --- | --- | --- | --- | --- | --- | --- | --- | --- | --- | --- | --- | --- | --- | --- | --- | --- | --- | --- | --- | --- | --- | --- | --- | --- | --- | --- | --- | --- | --- | --- | --- | --- | --- | --- | --- | --- | --- | --- | --- | --- | --- | --- | --- | --- | --- | --- | --- | --- | --- | --- | --- | --- | --- | --- | --- | --- | --- | --- | --- | --- | --- | --- | --- | --- | --- | --- | --- | --- | --- | --- | --- | --- | --- | --- | --- | --- | --- | --- | --- | --- | --- | --- | --- | --- | --- | --- | --- | --- | --- | --- | --- | --- | --- | --- | --- | --- | --- | --- | --- | --- | --- | --- | --- | --- | --- | --- | --- | --- | --- | --- | --- | --- | --- | --- | --- | --- | --- | --- | --- | --- | --- | --- | --- | --- | --- | --- | --- | --- | --- | --- | --- | --- | --- | --- | --- | --- | --- | --- | --- | --- | --- | --- | --- | --- | --- | --- | --- | --- | --- | --- | --- | --- | --- | --- | --- | --- | --- | --- | --- | --- | --- | --- | --- | --- | --- | --- | --- | --- | --- | --- | --- | --- | --- | --- | --- | --- | --- | --- | --- | --- | --- | --- | --- | --- | --- | --- | --- | --- | --- | --- | --- | --- | --- | --- | --- | --- | --- | --- | --- | --- | --- | --- | --- | --- | --- | --- | --- | --- | --- | --- | --- | --- | --- | --- | --- | --- | --- | --- | --- | --- | --- | --- | --- | --- | --- | --- | --- | --- | --- | --- | --- | --- | --- | --- | --- |

Table S5 | Samples that were predicted to have opposite gender from what was reported on the metadata but turned out to be copying errors.

| **Organism** | **Series** | **Samples** | **Reported gender** | **Prediction \| Reason** |
| --- | --- | --- | --- | --- |
| **Human** | GSE13205 | GSM333436 | 60 years old septic Male | Female \| The corresponding publication reports only a 60 years old female |
|  | GSE3526 | GSM80654, GSM80658, GSM80790 | All females | All males \| These samples were identified as duplicates. The original IDs report them as males |
|  | GSE38780 | GSM949391 | 17 years old female | Male \| The publication states one 17 years old male |
| **Mouse** | E-MEXP-733 | All samples | Mixed gender | All samples were strongly predicted as opposite gender \| Possible copying error |
|  | GSE25729 | GSM632001 | Male | Female \| Possible copying error |
|  | GSE1479 | Samples past E11 | All females | Mixed gender \| Gender differentiation in mice happens between E11 and E12 from which we had mixed gender predicted |

**Table S6 |** Supplementary equations for *ρ* to *Ζ* and *Z* to *ρ* transformations, and confidence intervals calculation:

| *ρ* to *Ζ* transformation using the inverse hyperbolic tangent function (arctanh): $Z_{\rho}=\frac{1}{2}\ln\left(\frac{1+\rho}{1-\rho}\right)=\mathrm{arctanh}(\rho)$   and its standard error:$SE_{Z}=\frac{1}{\sqrt{n-3}}$ | (Equation S1)  (Equation S2) |
| --- | --- |
| Confidence intervals calculation:     | (Equation S3) |
| *Z* to *ρ* transformation using the hyperbolic tangent function (tanh) $\rho=\frac{1-e^{2Z}}{1+e^{2Z}}=\tanh(Z)$  | (Equation S4) |
